# Global transcriptome and targeted metabolite analyses of roots reveal different defence mechanisms against *Ralstonia solanacearum* infection in two resistant potato cultivars

**DOI:** 10.3389/fpls.2022.1065419

**Published:** 2023-01-09

**Authors:** Jeny Jose, Csaba Éva, Zoltán Bozsó, Kamirán Áron Hamow, Zsófia Fekete, Attila Fábián, Zsófia Bánfalvi, László Sági

**Affiliations:** ^1^ Agricultural Institute, Centre for Agricultural Research, Martonvásár, Hungary; ^2^ Doctoral School of Plant Sciences, Hungarian University of Agriculture and Life Sciences, Gödöllő, Hungary; ^3^ Plant Protection Institute, Centre for Agricultural Research, Budapest, Hungary; ^4^ Institute of Genetics and Biotechnology, Hungarian University of Agriculture and Life Sciences, Gödöllő, Hungary

**Keywords:** disease resistance, lignification, phenolics, phenylpropanoid pathway, *Solanum tuberosum*, UPLC-MS

## Abstract

*Ralstonia solanacearum* (*Rs*), the causal agent of bacterial wilt disease in an unusually wide range of host plants, including potato (*Solanum tuberosum*), is one of the most destructive phytopathogens that seriously reduces crop yields worldwide. Identification of defence mechanisms underlying bacterial wilt resistance is a prerequisite for biotechnological approaches to resistance breeding. Resistance to *Rs* has been reported only in a few potato landraces and cultivars. Our *in vitro* inoculation bioassays confirmed that the cultivars ‘Calalo Gaspar’ (CG) and ‘Cruza 148’ (CR) are resistant to *Rs* infection. Comparative transcriptome analyses of CG and CR roots, as well as of the roots of an *Rs*-susceptible cultivar, ‘Désirée’ (DES), were carried out two days after *Rs* infection, in parallel with their respective noninfected controls. In CR and DES, the upregulation of chitin interactions and cell wall-related genes was detected. The phenylpropanoid biosynthesis and glutathione metabolism pathways were induced only in CR, as confirmed by high levels of lignification over the whole stele in CR roots six days after *Rs* infection. At the same time, *Rs* infection greatly increased the concentrations of chlorogenic acid and quercetin derivatives in CG roots as it was detected using ultra-performance liquid chromatography − tandem mass spectrometry. Characteristic increases in the expression of MAP kinase signalling pathway genes and in the concentrations of jasmonic, salicylic, abscisic and indoleacetic acid were measured in DES roots. These results indicate different *Rs* defence mechanisms in the two resistant potato cultivars and a different response to *Rs* infection in the susceptible cultivar.

## Introduction

*Ralstonia solanacearum* (*Rs*) is a generalist, (hemi)biotrophic phytopathogen that causes the destructive bacterial wilt disease or is maintained in approximately 400 host and nonhost reservoir species across more than 50 botanical families (Buddenhagen and Kelman, 1964; Hayward, 1991; Patil et al., 2012). Besides banana, eggplant, peanut, pepper, tobacco, and tomato, the primary crop affected by this pathogen worldwide is potato (*Solanum tuberosum* L.) (Elphinstone, 2005; Álvarez et al., 2010). The extent of global economic losses due to this pathogen is approximately 1 billion USD per year. The regional yield losses in potato crops range from 10% to 80% (reviewed by Kinyua et al., 2014; Kurabachew and Ayana, 2016; Karim et al., 2018; Savary et al., 2019). Based on economic and scientific importance, the *Rs* bacterium was ranked second among the top 10 plant pathogenic bacteria worldwide (Mansfield et al., 2012). This high position has been earned because of its lethality caused by wilting of the host plants, and because of its difficult eradication as a consequence of its prolonged survival and high persistence in the environment (van Elsas et al., 2000; Kong et al., 2014).

In Europe, *Rs* has been a quarantine pest since the early 1990s, and its quick spread in the continent is attributed to the global import of its many host plants, which have carried latent infection (Janse et al., 1998; Janse et al., 2004). The *Rs* strains causing the brown rot disease in potatoes in Europe belong to race 3 and biovar 2 (R3B2; Buddenhagen et al., 1962; Hayward, 1964), and to phylotype IIB and sequevar 1 (PIIB-1; Fegan and Prior, 2005). These strains are thought to have co-evolved along the Andes, down to Chile, in association with local *S. tuberosum* types that had become adapted to short or long days and to the cool-temperate climate (Hawkes, 1956; Spooner et al., 2005; Ames and Spooner, 2008; Hardigan et al., 2017).

Despite at least 2 million years of co-evolution between potato plants (Särkinen et al., 2013; Aversano et al., 2015) and the *Rs* bacterium (Marin et al., 2017; www.timetree.org), natural resistance or immunity among cultivated *S. tuberosum* genotypes has been very rare (Nielsen and Haynes, 1960; Jaworski et al., 1980). The success of this pathogen is associated with the concerted action of a broad range of virulence factors and effectors, many of which are transported *via* the type II and type III secretion systems into their host(s) (Genin and Denny, 2012; Coll and Walls, 2013; Peeters et al., 2013).

The path of the *Rs* pathogen from the soil up to the aerial tissues of host plants can be divided in four distinct phases (Planas-Marquès et al., 2020): root colonization, vertical movement to shoots, circular vascular (xylem) invasion, and radial spread into the cortex apoplast. Each of these phases are characterized by major changes in molecular, metabolic, and physiological processes both in the pathogen (Lowe-Power et al., 2018) as well as the host (Meline et al., 2022), which result in a wide range of compatible or incompatible outcomes. The plant root as the host-pathogen interface simultaneously represents a first major barrier and an entry point for the *Rs* bacterium (Xue et al., 2020). Root colonization is a relatively fast process: the initial external binding is followed within a few hours by invasion of the root cortex, then in 1-2 days the deeper vascular tissues, i.e., xylem vessels and tracheary elements (Vasse et al., 1995; McGarvey et al., 1999). By 3-4 days after infection, the bacteria can already be detected in the stem (Caldwell et al., 2017). This early phase of the infection is associated with a specific rewiring of developmentally and hormonally regulated metabolic pathways (Cao et al., 2020; Meline et al., 2022). The characterization of these processes is therefore instrumental in understanding plant resistance to *Rs* infection.

Numerous cross-breeding programs were initiated in the 1960s for the introgression of resistance to *Rs* from wild relatives, such as *S. phureja* (Thurston and Lozano, 1968; Sequeira and Rowe, 1969), *S. microdontum* (Tyagi et al., 1980), and *S. commersonii* (Carputo et al., 2009; Siri et al., 2009; Andino et al., 2022). Because of genetic distance and sexual incompatibility with resistant wild species, somatic hybridization was also attempted as a means by which to transfer *Rs* resistance from *S. phureja* (Fock et al., 2000), *S. stenotomum* (Fock et al., 2001; Fock et al., 2007), *S. chacoense* (Chen et al., 2013), *S. commersonii* (Laferriere et al., 1999; Kim-Lee et al., 2005), and even eggplant (*S. melongena*: Yu et al., 2013; Liu et al., 2016; Wang et al., 2020). However, due to the linkage drag of unfavourable traits (e.g., high-temperature sensitivity in the case of *S. phureja*), these efforts mainly resulted in *Rs*-resistant breeding lines (Park et al., 2016), but few commercially successful cultivars were produced (French et al., 1998; Patil et al., 2012; Huet, 2014; Muthoni et al., 2020).

Knowledge of the molecular mechanisms of *Rs* resistance is mainly based on studies performed with the model plant *Arabidopsis thaliana*. A known example of the first layer of defence against the *Rs* bacterium is that which is induced by the prokaryotic elongation factor-thermo unstable (EF-Tu or EF1A), the most abundant microbe-associated molecular pattern (Kunze et al., 2004), and a known elicitor in *Rs* (Lacombe et al., 2010; Fan et al., 2018). This elicitor activates a series of signalling events and defence reactions, collectively called pattern-triggered (or innate) immunity (PTI), which is initiated by EFR, the specific pattern-recognition receptor (PRR) kinase of EF-Tu in the Brassicaceae family (Zipfel et al., 2006). Since EF-Tu-triggered PTI is restricted to cruciferous plants (Kunze et al., 2004), this pathway of basal resistance is not available naturally in potatoes. The fact that transgenic transfer of the *Arabidopsis* EFR into tomato (Lacombe et al., 2010) and potato (Boschi et al., 2017) has conferred *Rs* resistance further supports the absence of a functional EFR ortholog in solanaceous plants (Zipfel et al., 2006). In addition to EFR, several receptors have already been characterized that take part in the detection of *Rs* and provide the host plant with resistance against this bacterium. These include the RRS1 and RPS4 in the *A. thaliana* ecotype ‘ND-1’ (Deslandes et al., 2003), ERECTA in the *A. thaliana* ecotype ‘Columbia’ (Godiard et al., 2003), Re-bw from the eggplant ‘E-31’ (Xiao et al., 2015), and AhRRS5 from peanut (Zhang et al., 2017). Ectopic overexpression of the AhRRS5 receptor conferred resistance to *Rs* in tobacco (Zhang et al., 2017).

Compared to *Arabidopsis*, relatively little is known about the resistance mechanisms of potato. One of the rare examples is reported by Narancio et al. (2013), who demonstrated that *Rs* infection of the damaged roots of pot-grown *S. commersonii* plants initiates a response in the stem as soon as 6 hours after the infection. At two days post-infection (dpi), pathways related to plant defence, such as ethylene (ET) and salicylic acid (SA) signalling were activated, while photosynthetic and certain transcription factor genes, including some *WRKY*s, were downregulated. Zuluaga et al. (2015) compared the transcriptome from root samples of an *Rs*-resistant and an *Rs*-susceptible *S. commersonii* accession at 3-4 dpi and found that the SA-related genes were downregulated in both accessions after pathogen infection, whereas the ET and jasmonic acid (JA) pathways were induced only in the susceptible accession. Cao et al. (2020) studied the early response of *S. tuberosum* to *Rs* in leaves at 2 dpi and found differentially expressed genes, including *LRR*s and *HSP*s, between the control and the *Rs*-infected sample, as well as genes involved in the biosynthesis of amino acids, plant hormone signal transduction, and starch and sucrose metabolism. Recently, Chen et al. (2021) silenced *StMKK1*, a mitogen-activated protein (MAP) kinase kinase in potato, which resulted in enhanced PTI and SA-related immune responses. The *StMKK1*-silenced lines developed almost no symptoms when tested at 5 dpi.

Based on the above findings, it is plausible that intrafamily PRRs and their interacting partners from resistant wild potatoes can be efficiently repurposed for engineering bacterial wilt resistance in potato cultivars. Alternatively, negative regulators of a step in the primary (basal) and secondary (systemic) resistance pathways can be used for targeted mutagenesis. Two main prerequisites for these approaches are the identification of *Rs*-resistant potato genotypes and their comprehensive and comparative molecular analysis to reveal essential information about the relevant defence mechanisms. To this end, nine potato accessions with reported resistance were collected and evaluated with an *in vitro* inoculation bioassay. The two most resistant cultivars were subjected to global transcriptomic and targeted metabolite analysis. The results of these analyses revealed the activation of different defence mechanisms in the two cultivars and led to the identification of several target genes for engineering resistance to *Rs*.

## Materials and methods

### Plant material and *in vitro* inoculation bioassay

Nine potato accessions known to be resistant or tolerant towards *Rs* (**Table 1**) were obtained from the gene banks of the USDA Agricultural Research Service (three accessions) and the International Potato Center (CIP, Peru: six accessions) to verify their disease resistance in comparison to the *Rs*-susceptible cultivar ‘Désirée’. The plants were maintained and propagated *in vitro* from single-node stem segments in test tubes in RM medium (MS medium without vitamins; Murashige and Skoog, 1962) supplemented with 20 g l^-1^ sucrose and 8 g l^-1^ agar at a constant 24°C with a 16 h photoperiod at a light intensity of 75 μmol m^-2^ s^-1^. Apical fragments with two-three leaves of 4-week-old plants were cut and placed in RM medium in a Phytatray vessel (Sigma-Aldrich, St. Luis, MI, USA, product no.: P5929). After 7-10 days, five plantlets with roots were transferred into rectangular Petri dishes (Greiner, Kremsmünster, Austria, product no.: 688102) containing the RM medium only in the lower half of the dishes, which was covered with a folded sterile filter paper to separate the roots from the medium. The Petri dishes, containing four-six plants, were placed in a vertical position and incubated at a constant 24°C with a 16 h photoperiod at a light intensity of 45-95 μmol m^-2^ s^-1^ for an additional 10-12 days before inoculation. In general, 20 plantlets per accession were tested for *Rs* inoculation.

**Table 1:** Potato accessions tested for *Rs* resistance.

Name	Accession ID	Year^a^	Pedigree (country of origin)	Taxonomy (resist. source)	Reference, source
‘Calalo Gaspar’	CIP 700670	1969	landrace (Peru: Junin, Mantaro)	*S. stenotomum* ssp. *stenotomum*	CIP database^b^, Martin (1979)
‘Cruza 148’	CIP 720118, PI 619136	1975	‘Montserrate’ × PI? (Mexico: Toluca)	*Solanum* hybrid (*S. demissum*)?	CIP database^c^, Martin (1979); Jackson (2012)
‘Kinga’ (BW-12.1)	CIP 377852.1	1988	‘Caxamarca’ × WRF-1923.1 (Peru)	*Solanum* hybrid (*S. phureja*)	Schmiediche (1983) French et al. (1998)
‘Monona’	AV 48	1964	‘Katahdin’ × ‘Chippewa’ (USA)	*S. tuberosum*	Stevenson et al. (1965) ECPD (2011)
‘Ontario’	AV 25	1946	‘Richter’s Jubel’ × USDA S44537 (USA)	*S. tuberosum*	Blodgett and Stevenson (1946); Jaworski et al. (1980)
MS-42.3	CIP 800928	1970s	? (Peru)	*Solanum* hybrid (*S. phureja*)	CIP (1979) Feng et al. (2003)
BW-6	CIP 381064.3	1980s	‘Kinga’ × AVRDC-1287.19 (Peru)	*Solanum* hybrid (*S. phureja*, …)	Schmiediche (1983) Tung et al. (1990)
BW-5	CIP 379695.4	1980s	CIP 377847.1 × CGN-69.1 (Peru)	*Solanum* hybrid (*S. demissum*)?	Schmiediche (1983)
BW-1.7	CIP 377831.7	1980s	‘Molinera’ × ‘Katahdin’ (Peru)	*Solanum* hybrid (*S. phureja*)	Schmiediche (1983) Montanelli et al. (1995)
‘Désirée’ (suscept.)	(CIP 800048)	1962	‘Urgenta’ × ‘Depesche’ (the Netherlands)	*S. tuberosum*	http://web.hzpc-holland.com/teeltbeschrijving/DESIREE

a Year of release (cultivars) or introduction (breeding lines).

b https://www.genesys-pgr.org/10.18730/8ZMW, http://germplasmdb.cip.cgiar.org/pages/report/CIP700670.html.

c https://www.genesys-pgr.org/10.18730/D5GM, http://germplasmdb.cip.cgiar.org/pages/report/CIP720118.html.

The *Rs* strain UW551 (R3B2; a wild-type geranium isolate, Swanson et al., 2005) is highly virulent on potatoes and is one of the references for bacterial wilt experiments (Siri et al., 2011; Hayes et al., 2017). This strain, transformed with the pDSK-GFPuv plasmid (Wang et al., 2007) containing the green fluorescent protein (GFP) reporter gene under the control of the constitutive *psbA* promoter, was grown on CPG medium (casamino acid 1 g l^-1^, peptone 10 g l^-1^, glucose 5 g l^-1^, agar 17 g l^-1^, pH 6.5) supplemented with 30 mg l^-1^ kanamycin and incubated at 28°C for 48 h, then regrown for another 48 h. Finally, bacteria were suspended at a concentration of 5-7 × 10^8^ CFU ml^-1^ (O.D._600_ = 0.8) in sterile deionized water. For inoculations, the plant roots were wounded by sterile scalpels approximately 1 cm above their tip and inoculated *via* pipette with 350 μl of bacterial suspension per plant. The rate of infection was observed at 1, 5, 7, 9, 12, 15, 19, and 21 days post-infection (dpi) in all the accessions. Photos were taken in visible light to assess the evolution of disease symptoms, as well as under UV light (iBright CL1500 Imaging System, Thermo Fisher Scientific) to monitor the spread of GFP-expressing bacteria within the plants.

### RNA isolation and transcriptome sequencing

Total RNA was extracted according to Stiekema et al. (1988) from the roots of 2-week-old *Rs*-inoculated potato plants (2 dpi) and uninoculated controls, which were grown in rectangular Petri-plates (see above) containing selected *Rs*-resistant cultivars ‘Calalo Gaspar’ and ‘Cruza 148’. ‘Désirée’ plants, both inoculated and un-inoculated, served in parallel as the *Rs*-susceptible control. The overall evaluation (quantity, purity, and integrity) of the total RNA samples was performed on agarose gels, by a NanoDrop spectrophotometer (Thermo Fisher Scientific, Waltham, MA, USA), and a 2100 Bioanalyzer (Agilent, Santa Clara, CA, USA). Half volumes of the samples (three biological replicates per treatment and cultivar, except for two replicates in ‘Cruza 148’) were used for cDNA synthesis after mRNA purification with poly-T oligo-attached magnetic beads. It was followed by paired-end, non-directional library construction (NEBNext Ultra RNA Library Prep Kit for Illumina; New England Biolabs, Ipswich, MA, USA), and, after quantification with a Qubit fluorometer (Thermo Fisher) and real-time PCR, by custom sequencing *via* an Illumina NovaSeq 6000 platform (Novogene, Nanjing, China).

The bioinformatic analysis included (i) quality control using fastp (Chen et al., 2018) to remove the adapter and poly-N sequences and low-quality data, which yielded high numbers of clean reads (**Supplementary Table 1**); (ii) mapping to the *S. tuberosum* group Phureja DM1–3 516 R44 (v6.1) reference genome sequence (Pham et al., 2020) using HISAT2 software (Kim et al., 2015) and annotation using reference annotation and novel gene prediction with StringTie (Pertea et al., 2015); (iii) gene expression quantification by featureCounts (Liao et al., 2014) measured in FPKM (Fragments Per Kilobase of transcript sequence per Million base pairs sequenced) as well as correlation analysis; (iv) differential expression analysis using DeSeq2 (Love et al., 2014) with a log2(FoldChange) cut-off value of ≥1 and the *p*-values conservatively adjusted for False Discovery Rate (FDR) according to Benjamini and Hochberg (1995); and (v) gene ontology (GO) enrichment and Kyoto Encyclopedia of Genes and Genomes (KEGG; Kanehisa and Goto, 2000) pathway analysis of differentially expressed genes (DEGs) using the ClusterProfiler R package (Wu et al., 2021). All these analyses were performed by automated Perl scripts by Novogene.

### Validation of the RNA-seq data by quantitative real-time PCR

The remaining halves of the RNA samples were treated with RQ1 RNase-free DNase (Promega, Madison, WI, USA) followed by random-primed reverse transcription to generate cDNA using the RevertAid First Strand cDNA Synthesis Kit (Thermo Fisher Scientific) according to the manufacturer’s instructions. The success of the DNase treatment and reverse transcription was checked by running standard PCRs on DNase-treated RNA as well as on cDNA as templates. The obtained cDNA was diluted four times before further use.

The expression of five target genes in the roots of the two *Rs*-resistant cultivars and *Rs*-susceptible control was characterized by quantitative PCR (qPCR). Members involved in the salicylic acid pathway, auxin signalling, and antioxidant pathways were tested, and two housekeeping genes, *β-TUBULIN* (Yin et al., 2016; accession No. Z33402 in NCBI) and *ELONGATION FACTOR 1α* (Nicot et al., 2005; accession No. AB061263.1), were amplified (for primer pairs see **Supplementary Table 2A**). Geometric means of the two housekeeping genes were used for normalization.

The qPCRs were carried out in triplicate in a 7500 Fast PCR System (Applied Biosystems, Waltham, ME, USA). A single reaction comprised 0.4 μl of cDNA, 1 μl of primer pair (10 μM), 5 μl of Fast SYBR Green master mix (Thermo Fisher Scientific), and 3.6 μl of water to constitute a total volume of 10 μl. The temperature profile included an initial denaturation step at 95°C for 20 sec, followed by 40 cycles of 5 s at 95°C and 30 s at 60°C. Expression levels of the studied genes were calculated using the 2^-ΔΔCt^ method (Livak and Schmittgen, 2001) with an efficiency correction step applied according to Pfaffl (2004). The data were analysed by ANOVA followed by Tukey’s *post-hoc* test.

### Metabolic screening by targeted ultra-performance liquid chromatography – tandem mass spectrometry

One gram of leaf and root tissues was taken from the two *Rs*-resistant cultivars ‘Calalo Gaspar’ and ‘Cruza 148’, and from the *Rs*-susceptible ‘Désirée’ at 0 dpi and 6 dpi. The samples were flash-frozen in liquid nitrogen and stored at -80°C until preparation by homogenization with liquid nitrogen, mortar, and pestle. Portions of 0.1 g homogenized frozen fresh weight (FW) plant material were transferred into 1.5-ml safety Eppendorf tubes and stored at -80°C until extraction. For extraction HPLC-grade chemicals and for elution UPLC-MS-grade acetonitrile were used (VWR, Radnor, PA, USA). Nonlabelled reference materials were purchased from Sigma-Aldrich (Darmstadt, Germany). Before extraction, samples were spiked with 2 ng of labelled [^2^H_6_](+)-*cis,trans*-abscisic acid (OlChemIm s.r.o. Olomouc, Czech Republic) as an internal standard. Samples were extracted with 500 µl of methanol:water (2:1), followed by 5 sec of vigorous vortexing; then, samples were shaken with a Spex (Metuchen, NJ, USA) Mini G 1600 in a cryo-cooled rack at 1500 rpm for 3 min. After centrifugation at 14,000 *g* and 4°C for 10 min, supernatants were collected, and the remaining pellets were re-extracted by repeating the extraction procedure once more. The respective supernatants were pooled to a final sample ratio of 0.1 g FW ml^-1^, filtered through a 0.22 µm PTFE syringe filter, then transferred to injection vials and submitted directly to analysis.

UPLC-MS/MS analysis and elution were carried out according to Vrhovsek et al. (2012) and Pál et al. (2019) with slight modifications. Briefly, separation was achieved on a Waters HSS T3 column (1.8 μm, 100 mm × 2.1 mm) using an Acquity I class UPLC system (Waters Corp., Milford, MA, USA). Gradient elution was used with 0.1 v% formic acid, both in water (A) and acetonitrile (B). Tandem mass spectrometric detection was performed on a Xevo TQ-XS (Waters) equipped with a UniSpray™ source operated in timed multiple reaction monitoring (MRM) mode as described by Pál et al. (2019). The respective MRM transitions were used for the quantitation of components detected above the limits of quantification (LoQ; listed in **Supplementary Table 3**) out of more than 120 target components.

### Metabolic data analysis

The online MetaboAnalyst 5.0 (www.metaboanalyst.ca) platform was used for performing the PCA (Principal Component Analysis), PLS-DA (Partial Least Squares-Discriminant Analysis) as well as generating VIP (Variable Importance in the Projection) plots, heat maps, and box plots. Except for box plots, the data were normalized with the median values and then log-transformed. The selection of a VIP cut-off value of 1.0 was based on the considerations by Chong and Jun (2005). Box plots were based on one-way (parametric) ANOVA with an adjusted *p*-value cut-off of 0.05 and Fisher’s LSD *post-hoc* analysis.

### Microtechniques and confocal laser scanning microscopy

Roots of noninfected and *Rs-*infected plants of all cultivars were collected at 6 dpi (n=5 per treatment and cultivar). A 10-mm long section was excised from all the roots at the middle region (approximately 5 cm from the root tip). Root sections were fixed in 60 mM phosphate buffer (pH 7.2) containing 4 w% formaldehyde, washed, dehydrated in a series of ethanol solutions, and gradually infiltrated with LR White resin (Agar Scientific Ltd., Stansted, UK). The resin was polymerized at 55°C for 48 h. Semi-thin (1-µm) cross sections were cut from the resin blocks, using an Ultracut-E microtome (Reichert-Jung, Heidelberg, Germany) and stained with safranin O (Searle Diagnostic, High Wycombe, UK) for cell wall, with Fast Green FCF (Sigma-Aldrich) applied as counterstain. The stained sections were mounted in 50 v% glycerol and examined with a TCS SP8 confocal laser scanning microscope (Leica Microsystems GmbH, Wetzlar, Germany). Safranin O was excited at 514 nm, and signals were detected at 600 to 720 nm. Fast Green FCF was excited at 633 nm, and signals were detected at 640 to 780 nm. Micrographs were taken using the Leica Advanced Fluorescence software v3.1.5.1638 (Leica Microsystems) with no further processing of the images.

## Results

### Screening for *Rs*-resistant potato cultivars

**Figure 1:**
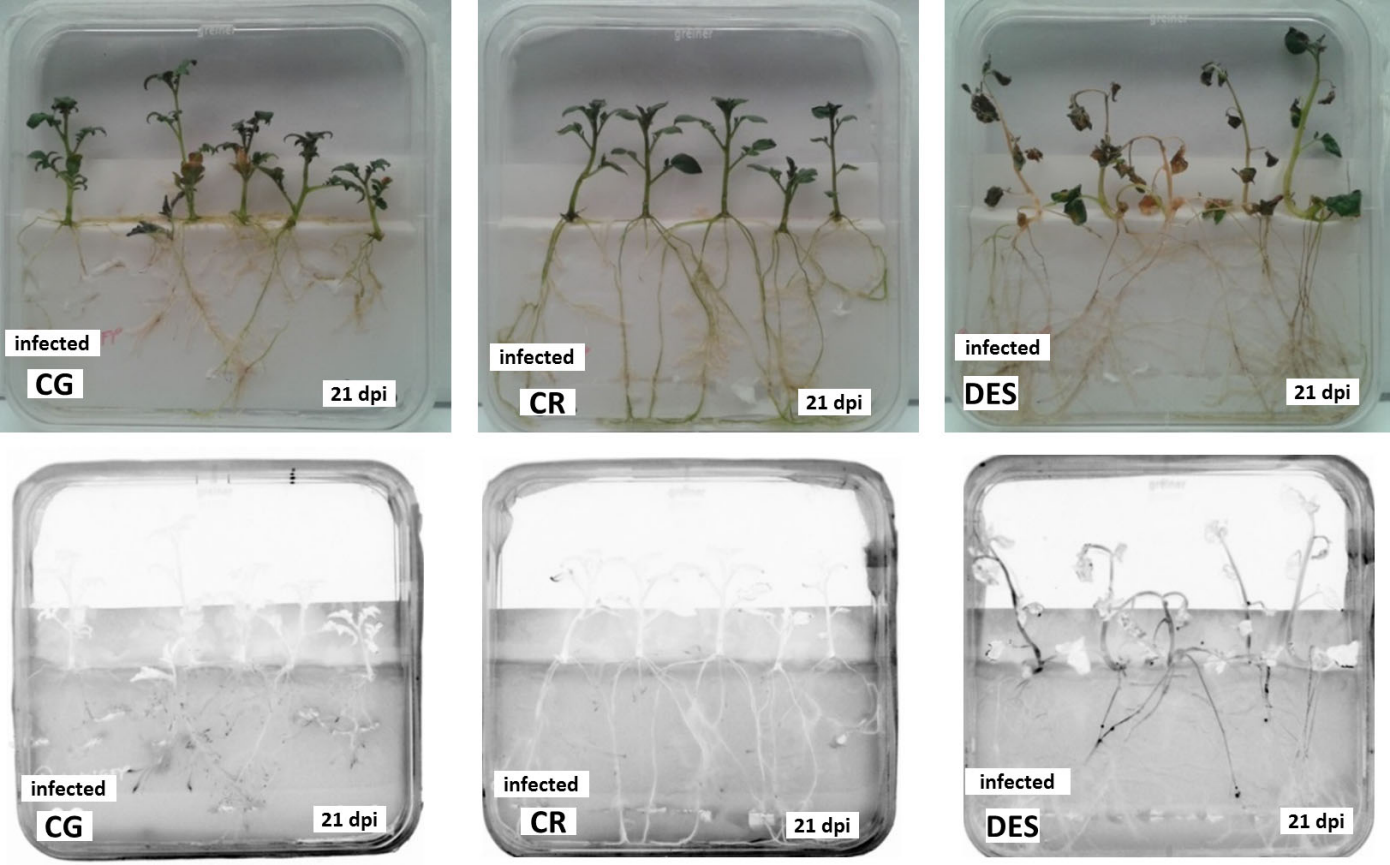
Visual evaluation of resistance to *Rs* infection (21 dpi) in the *in vitro* inoculation bioassay under visible light for the observation of wilting (upper row) and UV light for the detection of GFP-expressing *Rs* bacteria (lower row). CG, ‘Calalo Gaspar’; CR, ‘Cruza 148’; DES, ‘Désirée’.

Nine potato accessions (**Table 1**) were initially selected and propagated for testing *Rs* resistance in an *in vitro* inoculation bioassay. The highly virulent *Rs* strain UW551 (R3B2, PIIB-1) transformed with a GFP reporter gene-expressing plasmid was used for the *in vitro* inoculation of the potato plants. After a preliminary screening of the nine collected accessions and the *Rs-*susceptible commercial cultivar ‘Désirée’ as a control, the four highest-ranked accessions, ‘Calalo Gaspar’, ‘Cruza 148’, BW-5, and ‘Monona’, were selected for a repeated comparative bioassay. Two cultivars, ‘Calalo Gaspar’ and ‘Cruza 148’, survived at 100%, even at 21 dpi, compared to 32% survival of the control ‘Désirée’ (DES) (**Supplementary Table 4**). The two *Rs*-resistant cultivars remained green with minimal bacterial penetration, whereas the susceptible control wilted and the *Rs* bacteria were distributed throughout the plants (**Figure 1**), and the uninfected control plants grew vigorously (**Supplementary Figure 1**). Based on the results of this screening, ‘Calalo Gaspar’ (CG) and ‘Cruza 148’ (CR) were selected for further studies.

### Transcriptome analysis of *Rs*-infected potato roots

We performed a transcriptome analysis to reveal gene expression patterns in the *Rs*-resistant CG and CR and the *Rs*-susceptible DES plants at an early infection stage. Total RNA was isolated from noninfected and *Rs*-infected roots of *in vitro* plants at 2 dpi. A total of 16 samples were evaluated, composed of three biological replicates of CG and DES each and two biological replicates of CR. The data presented in **Supplementary Table 1** demonstrate that the purified RNA and the RNA sequencing was of good quality resulting in high RNA integrity numbers (RIN) and high frequencies of clean (between 95.8% and 97.6%) as well as uniquely mapped (85.4-88.8%) reads, respectively. The error rates were below 0.03% in all samples.

**Figure 2:**
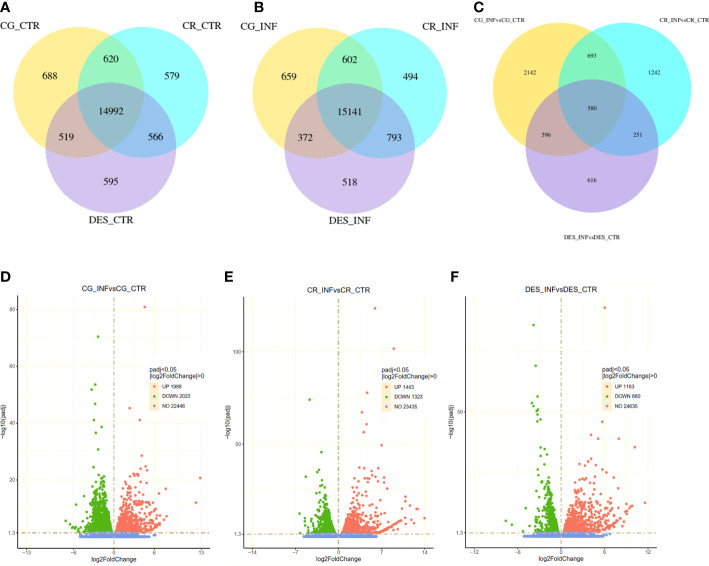
Number and distribution of coexpressed **(A, B)** and differentially expressed **(C-F)** genes in the roots of ‘Calalo Gaspar’ (CG), ‘Cruza 148’ (CR), and ‘Désirée’ (DES). **(A)** Coexpression of genes in noninfected control (CTR) roots at 2 dpi. **(B)** Coexpression of genes in *Rs*-infected (INF) roots at 2 dpi. **(C)** Differentially expressed genes upon *Rs* infection at 2 dpi. Volcano plots show the distribution of significantly (*p*adj<0.05) upregulated (red) and downregulated (blue) genes and those with unchanged expression (green) in the roots of CG **(D)**, CR **(E)**, and DES **(F)**.

Coexpression Venn diagrams showed that almost 15,000 genes were commonly expressed in noninfected roots of all cultivars, whereas the number of uniquely expressed genes was approximately 600-700 (**Figure 2A**). The *Rs* infection did not change these proportions substantially (**Figure 2B**). *Rs* infection, however, had a very different effect on each cultivar’s transcriptome, as only 580 differentially expressed genes (DEGs) in common were found, whereas 2,142, 1,242, and 616 unique DEGs were identified in CG, CR, and DES, respectively (**Figure 2C**). The total number of DEGs affected by *Rs* was 4,011 (1,988 up and 2,023 down) in CG, 2,766 (1,443 up, 1,323 down) in CR, and 2,043 (1,163 up, 880 down) in DES (**Figures 2D–F**).

**Figure 3:**
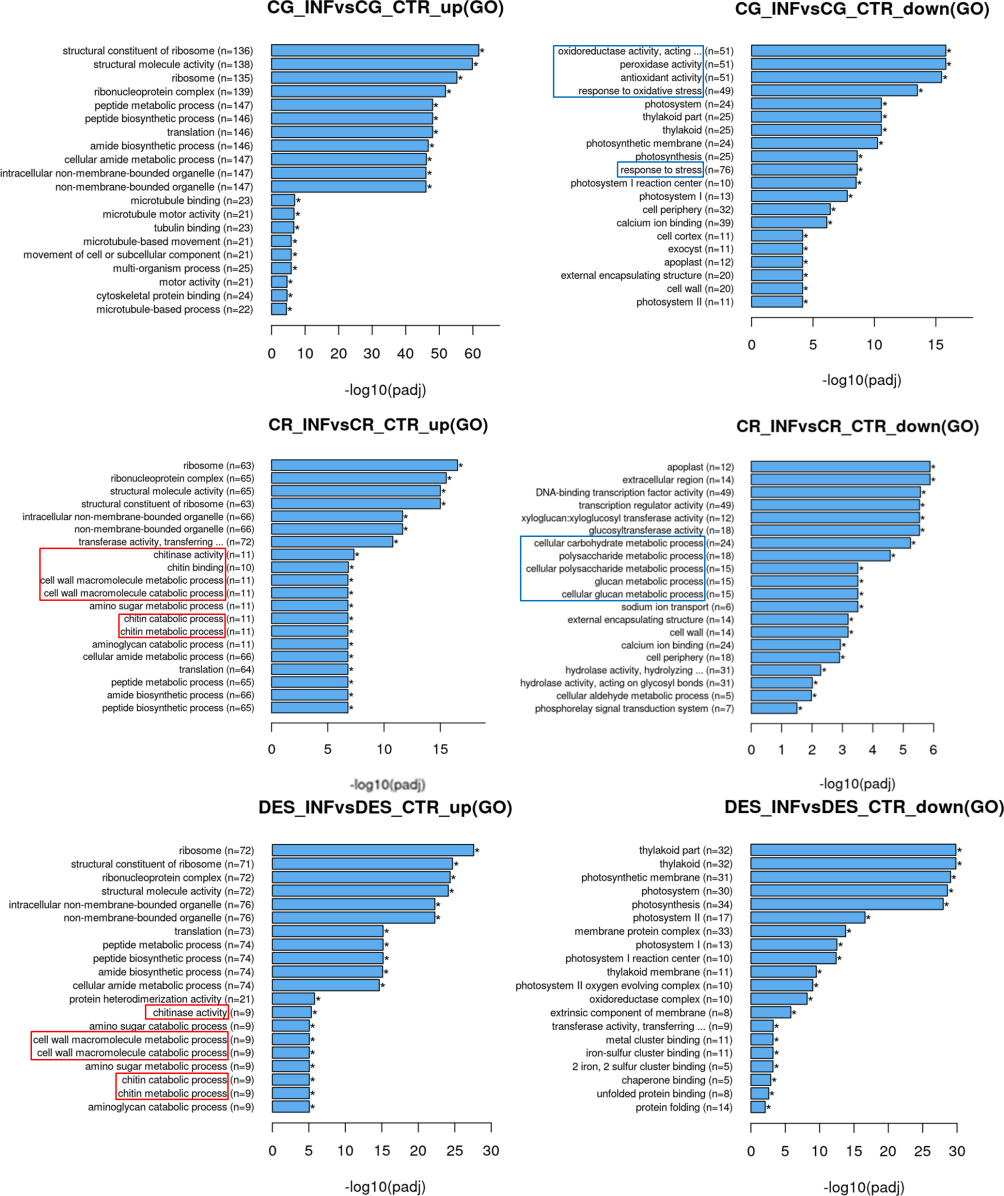
Upregulated and downregulated GOs upon *Rs* infection in the roots of ‘Calalo Gaspar’ (CG), ‘Cruza 148’ (CR), and ‘Désirée’ (DES). Asterisks indicate that all enrichments are significant. Categories with enrichments specific to each cultivar are boxed.

Gene ontology (GO) enrichment analysis (**Figure 3**) revealed that the ribosome-related genes and those associated with the ribonucleoprotein complex were upregulated in each cultivar. The photosystem was downregulated in CG and DES, but not in CR. The activity of photosynthesis-related genes in roots may seem surprising, however, under the *in vitro* conditions (see Methods) the roots were not in darkness. Moreover, downregulation of the photosystem may reflect the plant prioritizing defence against *Rs*. A large number of genes implicated in oxidative stress responses were downregulated by *Rs* infection in the CG cultivar. Both in CR and DES, induction of both cell wall and chitin metabolic as well as catabolic processes was detected. However, activation of these processes was accompanied by downregulation of the carbohydrate metabolism only in CR.

**Figure 4:**
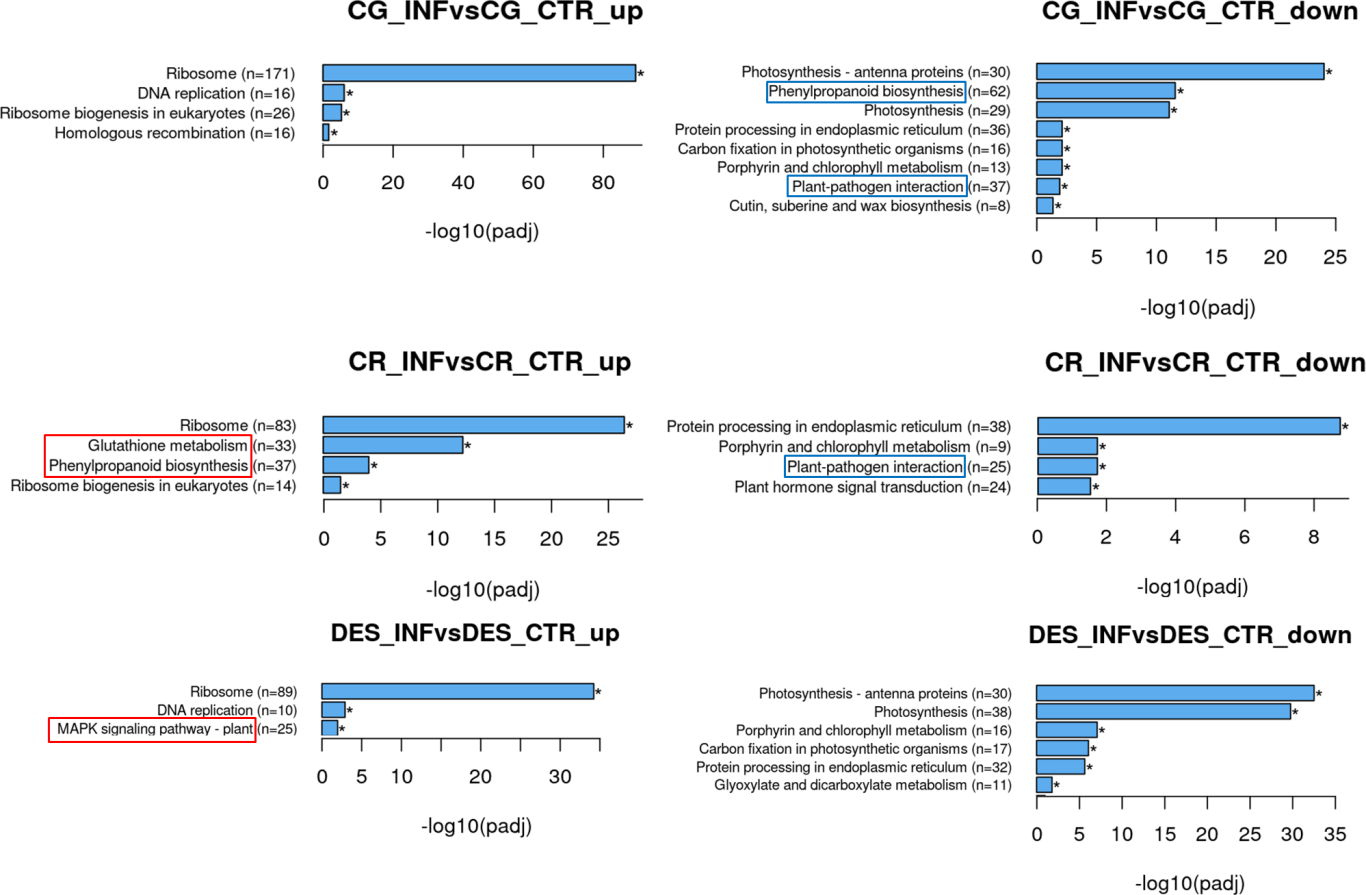
KEGG pathways significantly enriched upon *Rs* infection in the roots of ‘Calalo Gaspar’ (CG), ‘Cruza 148’ (CR), and ‘Désirée’ (DES). Asterisks indicate that the enrichments are significant. Pathways with enrichments specific to each cultivar are boxed.

The KEGG pathway analysis supported a part of GO enrichment, as phenylpropanoid and plant-pathogen interaction pathways were downregulated in CG. In contrast, in CR, the phenylpropanoid pathway was upregulated, while the plant-pathogen interaction pathway, as in CG, was downregulated (**Figure 4**). Further analysis of the phenylpropanoid biosynthesis genes that were downregulated in CG revealed that the majority, 45 out of the 62 genes identified, belonged to the family of peroxidases, and only 17 genes, including *PHENYLALANINE AMMONIA-LYASE* (*PAL*), were involved in different enzymatic steps of phenylpropanoid biosynthesis (**Supplementary Table 5**). In CR, 37 upregulated genes were sorted into the same category, out of which 27 encoded peroxidases (PODs) and 10 encoded different synthesis enzymes (**Supplementary Table 6**). Despite a large number of PODs with altered expression, only 8 were in common between the two sets of suppressed and induced genes in CG and CR, respectively. It is interesting to note that increased levels of lignin-forming anionic PODs and suberization-associated anionic PODs were detected in CR, which is in line with the result of GO analysis showing an increased intensity of cell wall metabolic and catabolic processes (**Figure 3**). As a unique feature, glutathione metabolism was upregulated in CR, including glutathione *S-*transferase, γ-glutamylcysteine synthetase, and ascorbate peroxidase genes.

Among the downregulated (log2FoldChange <1) genes in the plant-pathogen interaction pathway mainly the calcium-binding proteins were represented (30%) in CG, while no dominant family could be recognized in CR. Fifteen genes out of 37 and 25 (for CG and CR, respectively) were downregulated in both *Rs*-resistant cultivars, including *WRKY22* and *WRKY24* transcription factors and the heat shock protein genes *HSP90* and *HSP90-like* (**Supplementary Tables 7**, **8**). The relatively large proportion of the coregulated genes (41% and 60%) in the two *Rs*-resistant cultivars indicates that suppression of the corresponding processes may be important in the development of defence response to *Rs* infection.

In DES, in addition to general changes, the mitogen-activated protein (MAP) kinase signalling pathway was activated (**Figure 4**; **Supplementary Figures 2**, **3**). Moreover, the endochitinase gene *ChiB* was highly activated in each cultivar, as was *PR1* (*PATHOGENESIS-RELATED PROTEIN* 1) in DES and CG (**Supplementary Figure 2**). In contrast, several genes of the pathway were downregulated in the *Rs*-resistant cultivars, especially in CG (**Supplementary Figure 3**). In the DEG list, the *MAP KINASE KINASE KINASE A* (*MEKK1*) and *MAP KINASE 9* (*MAPK9*) were uniquely downregulated in CG, *MEKK7* was repressed in CR only and *MEKK EDR1* (*ENHANCED DISEASE RESISTANCE 1*) was downregulated in both *Rs*-resistant cultivars.

### Validation of RNA-seq data by real-time quantitative PCR

**Figure 5:**
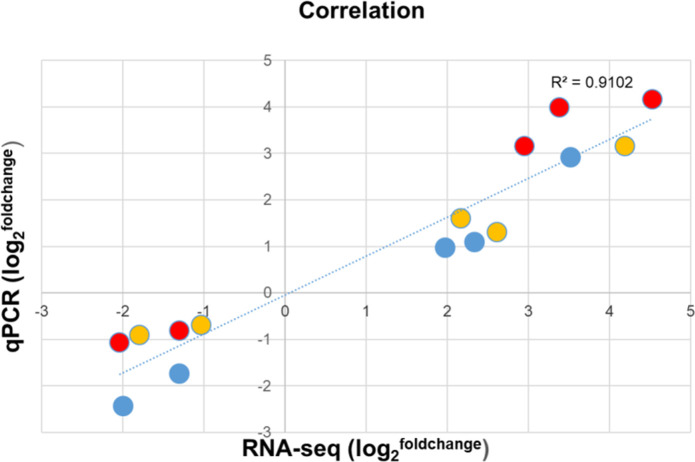
Differential expression of selected genes in the roots of noninfected and *Rs*-infected ‘Calalo Gaspar’ (CG), ‘Cruza 148’ (CR), and ‘Désirée’ (DES). The average fold change of biological replicates in expression levels detected by RNA-seq are compared to the fold change of the same biological replicates detected by RT-qPCR and are plotted on the x- and y-axis, respectively. Expression of *AGMATINE HYDROXYCINNAMOYLTRANSFERASE 1* (Soltu.DM.11G024290.1), *PATHOGENESIS-RELATED PROTEIN, STH-2* (Soltu.DM. 09G027710.1), *WOUND-INDUCED PROTEIN, WIN1* (Soltu.DM. 01G036460.1), *ABSCISIC ACID AND ENVIRONMENTAL STRESS-INDUCIBLE PROTEIN, TAS14* (Soltu.DM. 02G024670.1) and *L-ASCORBATE PEROXIDASE 2* (Soltu.DM. 09G006560.1) in CG (blue), CR (red) and DES (yellow).

To validate the results of RNA-seq data, five randomly picked genes, three of which were significantly upregulated and two of which were downregulated in each cultivar, were tested by RT-qPCR in the three potato cultivars. The expression trends and ranking of each tested gene obtained by RNA-seq and RT-qPCR were similar (**Supplementary Table 2B**) and resulted in a high correlation coefficient (*R^2^* = 0.9102) confirming the reliability of the transcriptome data (**Figure 5**).

### Targeted analysis of phenolics, flavonoids, and plant hormones

Plants synthesize a diversity of secondary metabolites, and it has long been known that plants with high concentrations of secondary metabolites are more resistant to biotic and abiotic stresses. Most defence compounds are phenolics synthesized in the phenylpropanoid pathway (Zaynab et al., 2018; Yadav et al., 2020). Besides phenolics, plant hormones, and especially ET, JA, SA, and abscisic acid (ABA) play major roles in crosstalk with growth-promoting compounds, in particular indoleacetic acid (IAA), to mediate plant defence responses against various stresses (Verma et al., 2016). Based on these findings, selected metabolites of the phenylpropanoid pathway as well as the plant hormones JA, SA, ABA, and IAA were quantified in CG, CR, and DES.

Root and leaf samples were collected from noninfected and *Rs*-infected potato plants at 6 dpi and analysed using UPLC-MS/MS. A total of 26 secondary metabolites and the four plant hormones listed above were selected for this study. However, not all the compounds were present in detectable amounts in both organs (**Supplementary Tables 9**, **10**).

**Figure 6:**
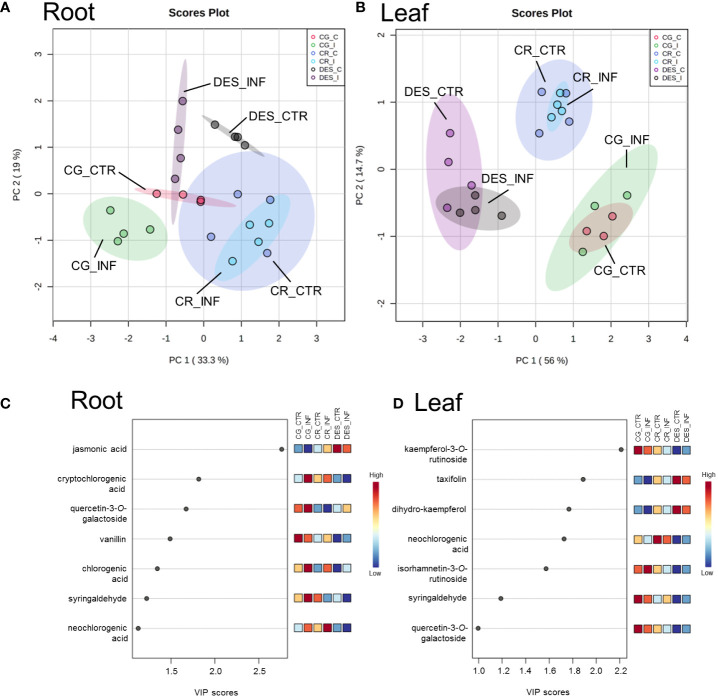
PCA (**A, B**) and VIP plots (**C, D**) showing the differences in the concentration of secondary metabolites between noninfected and *Rs*-infected ‘Calalo Gaspar’ (CG), ‘Cruza 148’ (CR), and ‘Désirée’ (DES) roots and leaves at 6 dpi. The data were obtained from four biological replicates from the roots of each cultivar and leaves of CG and DES and three biological replicates from the leaves of CR. Each biological replicate contained the roots and leaves of five plants. The raw data are available in **Supplementary Tables 9**, **10**. CTR, noninfected control; INF, infected.

To provide comparative interpretations and visualization of the metabolic differences, heatmaps were created (**Supplementary Figure 4**), and PCA and PLS-DA analyses were performed (**Figure 6**). Distinct profiles causing samples to cluster according to the cultivar were determined (**Figures 6A, B**). *Rs* infection changed the concentrations of several compounds in the roots, but to a lesser extent in CR than in CG and DES, as the PCA did not show a significant difference between the noninfected and *Rs*-infected CR roots (**Figure 6A**). *Rs* infection did not cause substantial changes in the levels of the analysed compounds in the leaves of any of the three potato cultivars (**Figure 6B**). To determine which compounds caused the major differences between the samples, VIP plots generated by PLS-DA were utilized. By selecting a value of 1.0 as the cut-off for the VIP values, seven metabolites in both the roots and leaves were found to cause the major differences between the samples (**Figures 6C, D**). These included chlorogenic acid and its two derivatives in roots, as well as kaempferol-3-*O*-rutinoside and dihydro-kaempferol in leaves.

**Figure 7:**
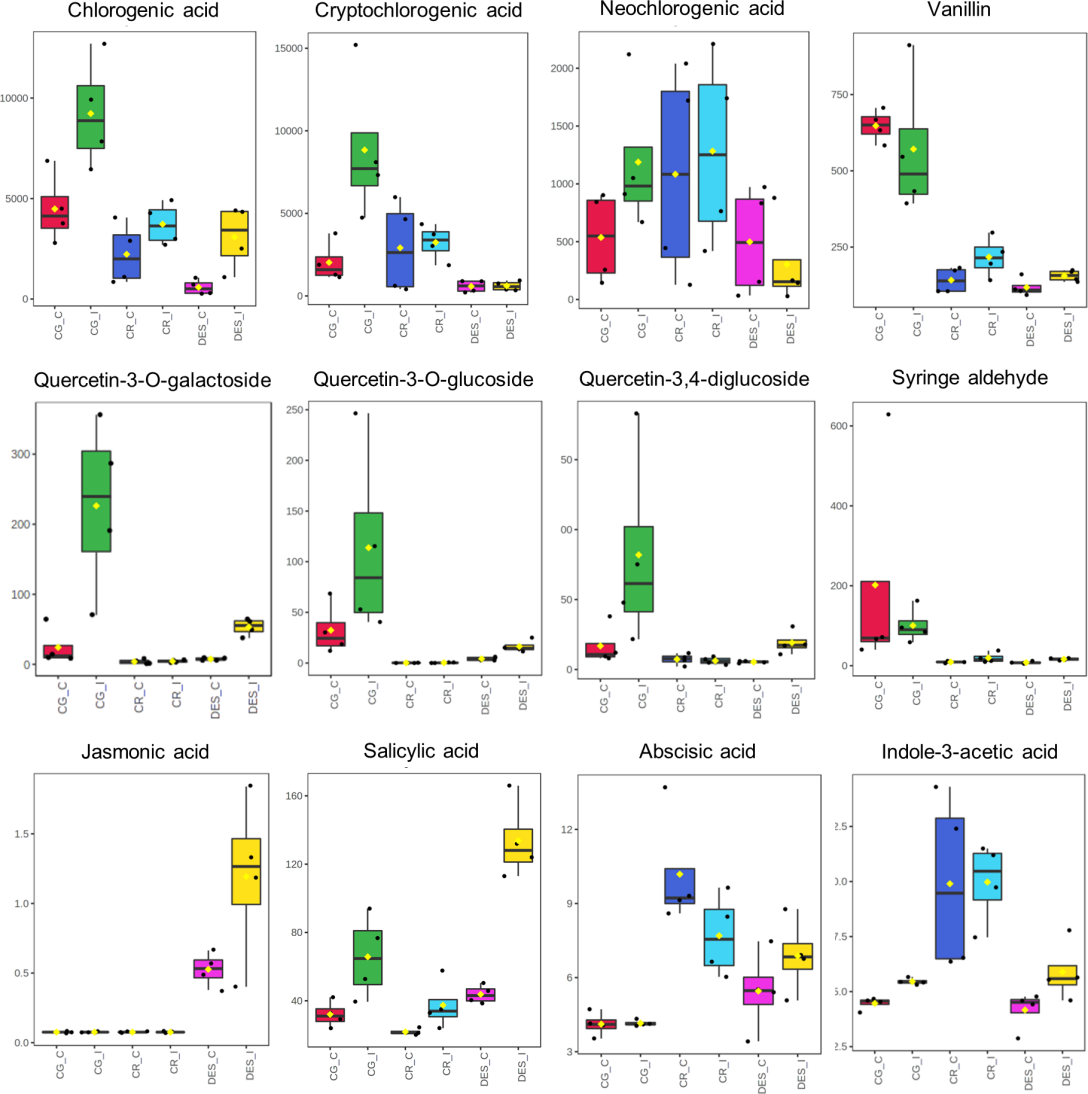
Box plots showing the concentration differences of eight selected secondary metabolites and four plant hormones between the noninfected and *Rs*-infected ‘Calalo Gaspar’, ‘Cruza 148’, and ‘Désirée’ roots at 6 dpi. The raw data are available in **Supplementary Tables 9**, **10**. CG_C, ‘Calalo Gaspar’ control; CG_I, ‘Calalo Gaspar’ infected; CR_C, ‘Cruza 148’ control; CG_I, ‘Cruza 148’ infected; DES_C, ‘Désirée’ control; DES_I, ‘Désirée’ infected.

A more detailed evaluation of the metabolic differences in roots with VIP score >1 and of the four plant hormones was carried out using box plots (**Figure 7**). Although the concentration of chlorogenic acid was higher in the *Rs*-resistant cultivars than in DES, it showed the same tendency to increase in the roots of each cultivar upon *Rs* infection. In contrast, the levels of cryptochlorogenic and neochlorogenic acids were significantly increased only in CG roots upon *Rs* infection. The concentrations of vanillin, syringaldehyde, and all three quercetin derivatives were much higher in CG roots than in the roots of the other two cultivars. *Rs* infection enhanced the levels of quercetin derivatives in CG and DES but not in CR. The PLS-DA identified JA as an important variable in the roots (**Figure 6C**). However, it was present, in low amounts only in DES roots. *Rs* infection increased the SA level in each cultivar. The ABA concentrations remained the same in CG, decreased in CR, and increased in DES roots. The IAA level was the highest in CR roots but was only increased by *Rs* infection in CG and DES.

### Lignification of *Rs*-infected potato roots

**Figure 8:**
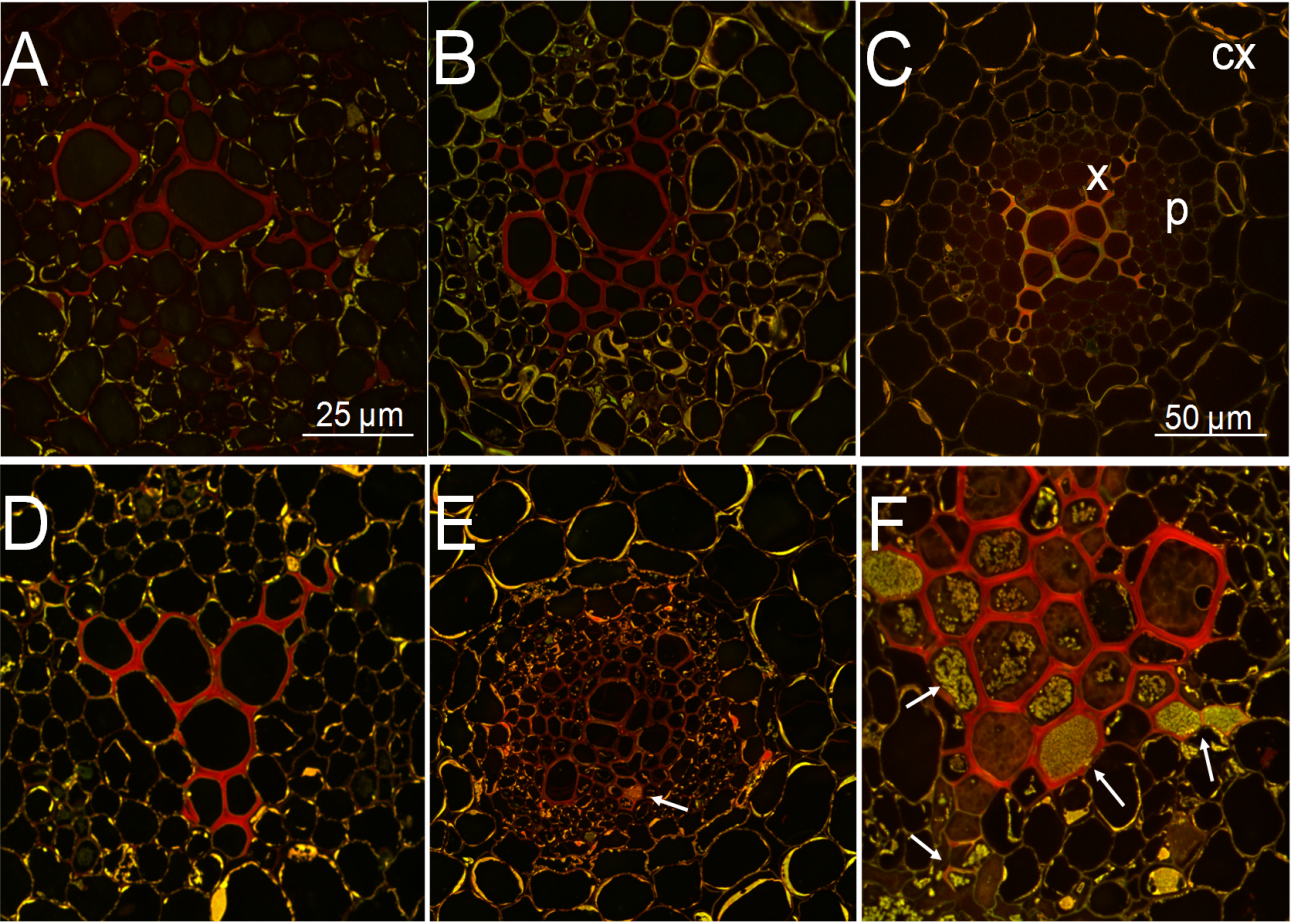
Confocal microscopy images of potato root cross-sections from noninfected control **(A-C)** and *Rs*-infected **(D-F)** plants at 6 dpi. Samples from 3-week-old, *in vitro* grown plants were stained with safranin for lignins and Fast Green for contrast. **(A)** ‘Calalo Gaspar’ control, **(B)** ‘Cruza 148’ control, **(C)** ‘Désirée’ control, **(D)** ‘Calalo Gaspar’ infected, **(E)** ‘Cruza 148’ infected, **(F)** ‘Désirée’ infected. cx, cortex; p; parenchyma; x, xylem; arrows, *Rs* bacteria.

GO analysis of transcriptome data indicated that *Rs* infection increased the cell wall and chitin metabolic and chitin catabolic processes in CR and DES roots (**Figure 3**). This prompted us to investigate by confocal microscopy the cell wall reinforcement in *Rs-*infected roots (6 dpi) compared to noninfected ones. Root cross-sections were therefore stained with safranin to detect the level of lignification (**Figure 8**). The stele, i.e. the central parts (primarily xylem) of the roots were lignified in all three cultivars, even without *Rs* infection (**Figures 8A–C**), but the area and intensity of red staining were the lowest in DES (**Figure 8C**). In addition to the central stele, the innermost layer of the cortex in the *Rs*-resistant CG cultivar also contained some lignin (**Figure 8A**). *Rs* infection did not change the extent of lignification in CG (**Figure 8D**), whereas it was enhanced over the whole stele in CR (**Figure 8E**) and even more strikingly in the xylem of DES (**Figure 8F**). A large amount of *Rs* bacteria was detected in DES (**Figure 8F**). Unlike in CG, the level of basal lignification in DES may not have been sufficient to prevent *Rs* invasion.

## Discussion

### Identification of *Rs*-resistant potato cultivars

To study the molecular mechanism of the resistance of potato plants against *Rs* infection, five cultivars and four breeding lines known to possess *Rs* resistance in the field (**Table 1**) were tested in an *in vitro* bioassay, and two cultivars, ‘Calalo Gaspar’ (CG) and ‘Cruza 148’ (CR), were found to be resistant under these conditions. ‘Calalo Gaspar’ is a traditional diploid landrace in Peru that belongs to the species *Solanum stenotomum*. This species was thought to be the progenitor of the cultivated potatoes and it is cross-compatible with *S. tuberosum* cultivars (Jackson et al., 1978; Gottschalk, 1984). It was reported to possess resistance against *Rs*, which has been transferred to *S. tuberosum* through breeding (Martin and French, 1985) and somatic hybridization (Fock et al., 2001). ‘Cruza 148’ is classified as ‘tuberosum type’ and originated from Toluca, Mexico. It was the first cultivated potato to demonstrate tolerance to bacterial wilt (Jackson et al., 1979). Since then, it has been regularly used as a moderately *Rs*-resistant variety check to screen breeding lines due to its durable low wilt percentage in standardized field experiments (Priou et al., 2001). Our *in vitro* inoculation tests confirmed these previous findings in the case of these two cultivars.

‘Kinga’ is a potato cultivar that has been commercially released in Madagascar (Hayward, 1994; Patil et al., 2012). ‘Monona’, a medium-early *S. tuberosum* cultivar, was produced in Texas and reported to have medium resistance against *Rs* (ECPD, 2011). Jaworski et al. (1980) found the cultivar ‘Ontario’ to be relatively resistant to *Rs* race 1 in Georgia (USA) in two consecutive years. Out of the four additional breeding lines (MS-42.3, BW-6, BW-5, and BW-1.7) included in the experiments, MS-42.3 was shown to express the antimicrobial peptide 1 (AP1), which is attributed to play a role in its resistance against *Rs* (Feng et al., 2003; Patil et al., 2012). However, none of these genotypes proved to be resistant in our *in vitro* inoculation bioassays. A plausible explanation for the lack of this response could be the fact that these genotypes were mainly tested with (tropical) race 1 *Rs* strains (Jaworski et al., 1980; Tung et al., 1990) and thus they may not be resistant to the race 3 UW551 strain that is virulent in cooler climate.

### Transcriptomic consequences of *Rs* infection

Several recent studies have identified genes related to defence against *Rs* in different plant species. In *Arabidopsis* roots, 2,698 DEGs were identified at 4 dpi, including several genes related to ABA, IAA, JA, and ET signalling cascades (Zhao et al., 2019). The role of the JA- and ET-mediated signalling in *Rs* resistance was found earlier in tomato as well, when an elicitin was infiltrated together with bacteria into the leaves (Kawamura et al., 2009). Using the same infiltration method, Baichoo and Jaufeerally-Fakim (2017) quantified the expression of marker genes of the JA, SA, and ET signalling pathways in *Lycopersicon cerasiforme*, *S. lycopersicum*, *S. commersonii*, and *S. tuberosum* and concluded that SA and ET signalling, but not JA signalling, play a significant role in defence against *Rs*. In contrast, French et al. (2018) suggested that roots mediate resistance to *Rs* through genome-wide transcriptomic changes that result in strong activation of defence genes and alteration of IAA pathways. In our *in vitro* experiment, the expression of thousands of genes was altered at 2 dpi in roots, however, no significant GO terms associated with any of the plant hormone signalling pathways were detected. In contrast, Zuluaga et al. (2015) found that SA-related genes were downregulated in both the roots of an *Rs*-resistant and an *Rs*-susceptible *S. commersonii* accession after pathogen infection and that the JA and ET pathways were induced in the susceptible accession. The contradiction could be the result of differences in the species, the experimental conditions (pot-grown plants vs. *in vitro* plants), and/or the time of sampling (2 dpi vs. 3-4 dpi). Nevertheless, we detected a concentration increase of each tested plant hormone (JA, SA, ABA, IAA) at 6 dpi in the infected roots of the *Rs*-susceptible cultivar ‘Désirée’ (DES). A possible explanation for the difference between our transcriptome and plant hormone results might be the difference in sampling time (2 dpi vs. 6 dpi). We note, however, that there is no consensus in terms of the role of plant hormones in defence against *Rs*. For example, the JA-insensitive *Arabidopsis* mutant *jar1-1* is susceptible to *Rs* infection (Hirsch et al., 2002), but the loss-of-function JA receptor mutation *coi1-1* enhances plant defence against *Rs* (Hernández-Blanco et al., 2007). Further, SA-deficient *NahG Arabidopsis* transgenic plants do not present detectable differences in wilt symptom development compared with the *Rs*-susceptible wild-type plants (Hirsch et al., 2002). However, overexpression of *NahG* (SA hydroxylase) restores the susceptibility of *Arabidopsis* to *Rs* in the *Rs*-resistant *wat1* (*walls are thin1*) mutant that is impaired in a gene required for secondary cell wall deposition (Denancé et al., 2013). The conclusion that can be drawn from our data is that increased concentrations of JA, SA, ABA, and IAA alone are not sufficient to prevent DES plants from *Rs-*induced wilting.

*Rs* infection upregulated the glutathione metabolism in CR roots. Peng et al. (2021) reported that glutathione metabolism was upregulated by *Rs* infection in eggplant roots and stems. An increase in glutathione *S-*transferase expression was detected also in *S. commersonii*, however, in both *Rs*-resistant and *Rs*-susceptible accessions (Zuluaga et al., 2015), while it was induced more than 10-fold in a *Rs*-resistant tomato cultivar in comparison with a susceptible cultivar (Ishihara et al., 2012). Transcriptome analysis of seedling roots of an *Rs*-resistant tobacco cultivar suggested that glutathione and flavonoids are probably the main substances conferring early resistance against *Rs* infection (Gao et al., 2019). This suggestion was supported by Li et al. (2021), who demonstrated that glutathione metabolism and phenylpropanoid pathways are the primary resistance pathways to *Rs* infection in tobacco. DEGs in glutathione metabolism were detected in the stems of a highly *Rs-*resistant tobacco variety as well (Pan et al., 2021). Glutathione is the most abundant antioxidant in cells and protects cell membranes and biomolecules from damage caused by reactive oxygen species (Dorion et al., 2021). Thus, it can contribute to the *Rs* resistance of the CR cultivar.

### Differential metabolic responses to *Rs* infection

Differences in CG, CR, and DES cultivars manifested not only at the transcriptomic level but also in the composition of phenolics in the roots, whereas no significant quantitative alterations of these compounds were detected in the leaves of any of the cultivars tested. This result indicates that at 6 dpi (the time of sampling) the effect of *Rs* infection did not reach the leaves at a level high enough to influence the concentration of the tested compounds. While the concentration of phenolics was substantially changed in CG and DES roots, it was only slightly influenced in CR. The increase in the concentrations of chlorogenic acid derivatives and quercetin derivatives was characteristic of CG. These compounds possess great antimicrobial and antioxidant potential and may contribute to protecting the plant from the harmful effect of *Rs* (Kabir et al., 2014). Chlorogenic acids are by far the most abundant phenolic compound in potato tubers and other organs (Friedman, 1997) and thus must have multiple important functions. Besides its well-known antimicrobial activity (Gebrechristos et al., 2020) and antioxidant capacity (Joly et al., 2021) chlorogenic acid is also involved in the synthesis of suberin (Valiñas et al., 2015) and lignin (Gamborg, 1967; Silva et al., 2019) biopolymers, the key compounds in cell wall fortification and reinforcement upon pathogen attack (Miedes et al., 2014; Höch et al., 2021; Wan et al., 2021).

The regulation of the synthesis of these metabolites, however, may be different in different species. For example, Wei et al. (2021), studying *Casuarina equisetifolia*, a tropical tree species, identified 18 flavonoids, including quercetin 3-*O*-glucoside, which accumulated differentially among three clonal groups (resistant, susceptible, and naturally infected by *Rs*), but the quercetin 3-*O*-glucoside level was lower in the infected *Rs*-resistant genotype than in the infected *Rs*-susceptible genotype. Dai et al. (2019) tested *Rs*-infected roots of mulberry at 1, 3, and 8 dpi and concluded that flavonoids may play important roles in bacterial wilt resistance in mulberry plants, as the key enzymes involved in flavonoid biosynthesis, such as chalcone synthase, chalcone isomerase, and flavonoid 3’-hydroxylase, were more significantly upregulated in the resistant than in the susceptible cultivar. We tested the expression of these genes by a manual search among DEGs of CG, CR, and DES and found only the genes encoding the different isoforms of chalcone synthase, which were all downregulated in CG and DES. In CR, one isoform was upregulated, while the other isoform was downregulated (data not shown).

The *Rs* infection enhanced the lignification over the whole stele in CR roots, as detected by confocal microscopy. Lignin is one of the most important secondary metabolites and is produced by the phenylalanine/tyrosine metabolic pathway in plant cells. The biosynthesis pathway of lignin in higher plants starts with PAL and ends with POD and laccase (reviewed by Liu et al., 2018). In line with this, the expression of two *PAL* transcript variants and those of several *POD*s were significantly upregulated in CR roots upon *Rs* infection. Lignin is a major player in the response of plants to various biotic and abiotic stresses, and MYB transcription factors and microRNAs are involved in the control of stress lignin deposition (reviewed by Cesarino, 2019). In our experiment, a large number of *MYB* DEGs (30 to 55) were identified in each cultivar. In line with its unperturbed lignification, the majority of *MYB*s (40/55, 73%) were slightly downregulated in CG, while in CR and DES a characteristic increase in the number of upregulated *MYB*s balanced the distribution (**Supplementary Table 11**).

The GO analysis showed an increased intensity of cell wall metabolic and catabolic processes in both CR and DES. The cell wall is the first physical layer of plant defence against pathogens, as demonstrated in *Arabidopsis* mutants impaired in cell wall-cellulose synthesis that alterations of the primary and secondary cell wall formation conferred resistance against vascular pathogens, including *Rs* (Menna et al., 2021). The underlying mechanism is not directly related to the modified cell wall structure; instead, it involves distinct localized channelling steps that activate defence pathways mediated by the plant hormones ABA (Hernández-Blanco et al., 2007; Feng et al., 2012) and IAA (Denancé et al., 2013).

### Miscellaneous pathways in *Rs* resistance

WRKY transcription factors are critical players in modulating plant resistance to phytopathogens and were also reported to function in plant defence to *Rs*. WRKY52/RRS1 confers resistance towards several strains of *Rs via* its interaction with the bacterial type III effector PopP2 (Deslandes et al., 2003). On the other hand, other *WRKY*s, such as *WRKY7*, *11, 17, 18, 40*, and *60* act as negative regulators of basal resistance in *A. thaliana* (Journot-Catalino et al., 2006; Kim et al., 2006; Wang et al., 2006). We found that *WRKY22* and *WRKY24* were downregulated in both CG and CR. In *Arabidopsis*, rice, and tobacco, the expression of *WRKY22* is upregulated by bacterial pathogens and contributes to host resistance (Abbruscato et al., 2012; Hsu et al., 2013; Ramos et al., 2021). WRKY22 also acts as a positive regulator in the pepper response to *Rs* (Hussain et al., 2018). In contrast, WRKY22 enhances susceptibility to citrus canker caused by the bacterium *Xanthomonas citri* (Long et al., 2021). WRKY44 is a positive regulator of early immune response in rice (Sheikh et al., 2021). Thus, we can conclude that depending on the pathogen and plant species, WRKYs can have opposite roles in mediating immunity to bacterial pathogens. Since both *WRKY22* and *WRKY24* were downregulated in CG and CR upon *Rs* infection, their role might be negative with respect to pathogen defence in potato.

The MAP kinase (MAPK) signalling pathway was activated in the *Rs*-susceptible cultivar DES. MAPK cascades are encoded by large multigenic families. The individual members of the family possess different roles in developmental processes as well as in responses to abiotic and biotic stresses. MAPKs are targets of virulent and avirulent effectors (reviewed by Lang and Colcombet, 2020). Chen et al. (2021) demonstrated that silencing the *StMKK1* encoding a MAP kinase kinase in potato results in almost symptomless *Rs* infection. We found *MAPK9 MEKK1* in CG, *MEKK7* in CR, and *MEKK EDR1* in both cultivars to be downregulated suggesting that reducing the activity of MAPKs may be a good strategy to alleviate losses caused by *Rs*.

Differences between the transcriptomic and metabolic responses of CG, CR, and DES to *Rs* infection are summarized in **Supplementary Figure 5**.

## Conclusion

The *in vitro* bioassay used in our experiment supported the previous findings that the *S. stenotomum* landrace ‘Calalo Gaspar’ (CG) and the potato cultivar ‘Cruza 148’ (CR) are resistant to *Rs* infection. In comparison to the *Rs*-susceptible cultivar ‘Désirée’ (DES), one-two thousand genes (out of the ca. 15,000 in common) were differentially expressed in the *Rs*-infected roots of the two resistant cultivars. In CR, the phenylpropanoid pathway and glutathione metabolism were upregulated, and increased activity of cell wall metabolic and catabolic processes was detected at the transcriptome level, which was associated with enhanced lignification in the stele. Increased concentrations of chlorogenic acid derivatives and quercetin derivatives were characteristic of *Rs*-infected CG roots. Phenolic compounds and glutathione have antimicrobial and antioxidant properties, respectively, and lignification can be a physical barrier to microbial invasion. Thus, both strategies can be successful against *Rs.* Considering that chlorogenic acid also functions as an intermediate in lignin biosynthesis, there might be a common regulation step in the two cultivars that results in *Rs* resistance.

## Data availability statement

The datasets presented in this study can be found in online repositories. The names of the repository/repositories and accession number(s) can be found below: Gene Expression Omnibus (https://www.ncbi.nlm.nih.gov/geo/info/linking.html) under accession no. GSE 211973.

## Author contributions

ZsB and LS conceived and designed the study, performed data analysis, wrote, revised, and finalised the manuscript. JJ and CE performed the molecular and bioinformatic analyses. ZoB performed the *in vitro* inoculation bioassays. KH carried out the metabolic screening. ZF and AF executed bioinformatic and microscopic analysis (with JJ), respectively. All authors contributed to the article and approved the submitted version.

## References

[B1] Abbruscato P. Nepusz A. Mizzi L. Del Corvo M. Morandini P. Fumasoni I.. (2012). *OsWRKY22*, a monocot *WRKY* gene, plays a role in the resistance response to blast. Mol. Plant Pathol. 13, 828–841. doi: 10.1111/j.1364-3703.2012.00795.x 22443363 PMC6638809

[B2] Álvarez B. Biosca E. G. López M. M. (2010). “On the life of ralstonia solanacearum, a destructive bacterial plant pathogen,” in Current research, technology and education topics in applied microbiology and microbial biotechnology. Ed. Méndez-Vilas A. (Badajoz: Formatex), 267–279.

[B3] Ames M. Spooner D. M. (2008). DNA From herbarium specimens settles a controversy about origins of the European potato. Am. J. Bot. 95, 252–257. doi: 10.3732/ajb.95.2.252 21632349

[B4] Andino M. Gaiero P. González-Barrios P. Galván G. Vilaró F. Speranza P. (2022). Potato introgressive hybridisation breeding for bacterial wilt resistance using *Solanum commersonii* dun. as donor: Genetic and agronomic characterisation of a backcross 3 progeny. Potato Res. 65, 119–136. doi: 10.1007/s11540-021-09512-1

[B5] Aversano R. Contaldi F. Ercolano M. A. Grosso V. Iorizzo M. Tatino F.. (2015). The *Solanum commersonii* genome sequence provides insights into adaptation to stress conditions and genome evolution of wild potato relatives. Plant Cell 27, 954–968. doi: 10.1105/tpc.114.135954 25873387 PMC4558694

[B6] Baichoo Z. Jaufeerally-Fakim Y. (2017). *Ralstonia solanacearum* upregulates marker genes of the salicylic acid and ethylene signaling pathways but not those of the jasmonic acid pathway in leaflets of *Solanum* lines during early stage of infection. Eur. J. Plant Pathol. 147, 615–625. doi: 10.1007/s10658-016-1030-7

[B7] Benjamini Y. Hochberg Y. (1995). Controlling the false discovery rate: a practical and powerful approach to multiple testing. J. R. Statist. Soc B 57, 289–300. doi: 10.1111/j.2517-6161.1995.tb02031.x

[B8] Blodgett F. M. Stevenson F. J. (1946). The new scab-resistant potatoes, Ontario, Seneca and Cayuga. Am. Potato J. 23, 315–329. doi: 10.1007/BF02888645

[B9] Boschi F. Schvartzman C. Murchio S. Ferreira V. Siri M. I. Galván G. A.. (2017). Enhanced bacterial wilt resistance in potato through expression of *Arabidopsis* EFR and introgression of quantitative resistance from *Solanum commersonii* . Front. Plant Sci. 8. doi: 10.3389/fpls.2017.01642 PMC5627020 29033958

[B10] Buddenhagen I. W. Kelman A. (1964). Biological and physiological aspects of bacterial wilt caused by *Pseudomonas solanacearum* . Annu. Rev. Phytopathol. 2, 203–230. doi: 10.1146/annurev.py.02.090164.001223

[B11] Buddenhagen I. Sequeira L. Kelman A. (1962). Designation of races in *Pseudomonas solanacearum* . Phytopathology 52, 726.

[B12] Caldwell D. Kim B.-S. Iyer-Pascuzzi A. S. (2017). *Ralstonia solanacearum* differentially colonizes roots of resistant and susceptible tomato plants. Phytopathology 107, 528–536. doi: 10.1094/PHYTO-09-16-0353-R 28112595

[B13] Cao W. Gan L. Shang K. Wang C. Song Y. Liu H.. (2020). Global transcriptome analyses reveal the molecular signatures in the early response of potato (*Solanum tuberosum* l.) to *Phytophthora infestans*, *Ralstonia solanacearum*, and potato virus y infection. Planta 252, 1–13. doi: 10.1007/s00425-020-03471-6 32955625

[B14] Carputo D. Aversano R. Barone A. Di Matteo A. Iorizzo M. Sigillo L.. (2009). Resistance to *Ralstonia solanacearum* of sexual hybrids between *Solanum commersonii* and *S. tuberosum* . Am. J. Potato Res. 86, 196–202. doi: 10.1007/s12230-009-9072-4

[B15] Cesarino I. (2019). Structural features and regulation of lignin deposited upon biotic and abiotic stresses. Curr. Opin. Biotech. 56, 209–214. doi: 10.1016/j.copbio.2018.12.012 30684783

[B16] Chen L. Guo X. Xie C. He L. Cai X. Tian L.. (2013). Nuclear and cytoplasmic genome components of *Solanum tuberosum* + *S. chacoense* somatic hybrids and three SSR alleles related to bacterial wilt resistance. Theor. Appl. Genet. 126, 1861–1872. doi: 10.1007/s00122-013-2098-5 23580089

[B17] Chen X. Wang W. Cai P. Wang Z. Li T. Du Y. (2021). The role of the MAP kinase–kinase protein StMKK1 in potato immunity to different pathogens. Hortic. Res. 8, 117. doi: 10.1038/s41438-021-00556-5 34059659 PMC8167122

[B18] Chen S. Zhou Y. Chen Y. Gu J. (2018). Fastp: an ultra-fast all-in-one FASTQ preprocessor. Bioinformatics 34, i884–i890. doi: 10.1093/bioinformatics/bty560 30423086 PMC6129281

[B19] Chong I.-G. Jun C.-H. (2005). Performance of some variable selection methods when multicollinearity is present. Chemometr. Intell. Lab. 78, 103–112. doi: 10.1016/j.chemolab.2004.12.011

[B20] CIP (1979). “Control of important bacterial diseases of potatoes,” in Annual report 1979 (Lima: International Potato Center), 21–27. Available at: https://hdl.handle.net/10568/109496.

[B21] Coll N. S. Walls M. (2013). Current knowledge on the *Ralstonia solanacearum* type III secretion system. Microb. Biotechnol. 6, 614–620. doi: 10.1111/1751-7915.12056 23617636 PMC3815929

[B22] Dai F. Luo G. Wang Z. Kuang Z. Huang J. Tang C. (2019). Possible involvement of flavonoids in response of mulberry (*Morus alba* l.) to infection with *Ralstonia solanacearum* . Eur. J. Hortic. Sci. 84, 161–170. doi: 10.17660/eJHS.2019/84.3.6

[B23] Denancé N. Ranocha P. Oria N. Barlet X. Rivière M. P. Yadeta K. A.. (2013). Arabidopsis *wat1* (*walls are thin1*)-mediated resistance to the bacterial vascular pathogen, *Ralstonia solanacearum*, is accompanied by cross-regulation of salicylic acid and tryptophan metabolism. Plant J. 73, 225–239. doi: 10.1111/tpj.12027 22978675

[B24] Deslandes L. Olivier J. Peeters N. Feng D. X. Khounlotham M. Boucher C.. (2003). Physical interaction between RRS1-r, a protein conferring resistance to bacterial wilt, and PopP2, a type III effector targeted to the plant nucleus. Proc. Natl. Acad. Sci. U.S.A. 100, 8024–8029. doi: 10.1073/pnas.1230660100 12788974 PMC164706

[B25] Dorion S. Ouellet J. C. Rivoal J. (2021). Glutathione metabolism in plants under stress: beyond reactive oxygen species detoxification. Metabolites 11, 641. doi: 10.3390/metabo11090641 34564457 PMC8464934

[B26] ECPD (2011). “Monona,” in The European cultivated potato database (Science and Advice for Scottish Agriculture). Available at: https://www.europotato.org/varieties/view/Monona.

[B27] Elphinstone J. G. (2005). “The current bacterial wilt situation: A global overview,” in Bacterial wilt disease and the ralstonia solanacearum species complex. Eds. Allen C. Prior P. Hayward,. A. C. (St. Paul: APS Press), 9–28.

[B28] Fan X. Zhao Z. Li Y. Zhuo T. Hu X. Zou H. (2018). The EF-tu epitope elf26 of *Ralstonia solanacearum* can promote resistance to bacterial wilt disease in *Nicotiana* species. Can. J. Plant Pathol. 40, 387–398. doi: 10.1080/07060661.2018.1483968

[B29] Fegan M. Prior P. (2005). “How complex is the ‘Ralstonia solanacearum species complex,” in Bacterial wilt disease and the ralstonia solanacearum species complex. Eds. Allen C. Prior P. Hayward A. C. (St. Paul: APS Press), 449–461.

[B30] Feng D. X. Tasset C. Hanemian M. Barlet X. Hu J. Trémousaygue D.. (2012). Biological control of bacterial wilt in *Arabidopsis thaliana* involves abscissic acid signalling. New Phytol. 194, 1035–1045. doi: 10.1111/j.1469-8137.2012.04113.x 22432714

[B31] Feng J. Yuan F. Gao Y. Liang C. Xu J. Zhang C.. (2003). A novel antimicrobial protein isolated from potato (*Solanum tuberosum*) shares homology with an acid phosphatase. Biochem. J. 376, 481–487. doi: 10.1042/BJ20030806 12927022 PMC1223772

[B32] Fock I. Collonnier C. Lavergne D. Vaniet S. Ambroise A. Luisetti J.. (2007). Evaluation of somatic hybrids of potato with *Solanum stenotomum* after a long-term *in vitro* conservation. Plant Physiol. Biochem. 45, 209–215. doi: 10.1016/j.plaphy.2007.02.004 17400465

[B33] Fock I. Collonnier C. Luisetti J. Purwito A. Souvannavong V. Vedel F.. (2001). Use of *Solanum stenotomum* for introduction of resistance to bacterial wilt in somatic hybrids of potato. Plant Physiol. Biochem. 39, 899–908. doi: 10.1016/S0981-9428(01)01307-9

[B34] Fock I. Collonnier C. Purwito A. Luisetti J. Souvannavong V. Vedel F.. (2000). Resistance to bacterial wilt in somatic hybrids between *Solanum tuberosum* and *Solanum phureja* . Plant Sci. 160, 165–176. doi: 10.1016/S0168-9452(00)00375-7 11164589

[B35] French E. R. Anguiz R. Aley P. (1998). “The usefulness of potato resistance to ralstonia solanacearum, for the integrated control of bacterial wilt,” in Bacterial wilt disease - molecular and ecological aspects. Eds. Prior P. Allen C. Elphinstone J. (Berlin-Heidelberg: Springer), 381–385.

[B36] French E. Kim B.-S. Rivera-Zuluaga K. Iyer-Pascuzzi A. S. (2018). Whole root transcriptomic analysis suggests a role for auxin pathways in resistance to *Ralstonia solanacearum* in tomato. Mol. Plant-Microbe Int. 31, 432–444. doi: 10.1094/MPMI-08-17-0209-R 29153016

[B37] Friedman M. (1997). Chemistry, biochemistry, and dietary role of potato polyphenols. a review. J. Agric. Food Chem. 45, 1523–1540. doi: 10.1021/jf960900s

[B38] Gamborg O. L. (1967). Aromatic metabolism in plants: V. the biosynthesis of chlorogenic acid and lignin in potato cell cultures. Can. J. Biochem. 45, 1451–1457. doi: 10.1139/o67-171 6048392

[B39] Gao W. Chen R. Pan M. Tang W. Lan T. Huang L.. (2019). Early transcriptional response of seedling roots to *Ralstonia solanacearum* in tobacco (*Nicotiana tabacum* l.). Eur. J. Plant Pathol. 155, 527–536. doi: 10.1007/s10658-019-01788-x

[B40] Gebrechristos H. Y. Ma X. Xiao F. He Y. Zheng S. Oyungerei G.. (2020). Potato peel extracts as an antimicrobial and potential antioxidant in active edible film. Food Sci. Nutr. 8, 6338–6345. doi: 10.1002/fsn3.1119 33312520 PMC7723200

[B41] Genin S. Denny T. P. (2012). Pathogenomics of the *Ralstonia solanacearum* species complex. Annu. Rev. Phytopathol. 50, 67–89. doi: 10.1146/annurev-phyto-081211-173000 22559068

[B42] Godiard L. Sauviac L. Torii K. U. Grenon O. Mangin B. Grimsley N. H.. (2003). ERECTA, an LRR receptor-like kinase protein controlling development pleiotropically affects resistance to bacterial wilt. Plant J. 36, 353–365. doi: 10.1046/j.1365-313X.2003.01877.x 14617092

[B43] Gottschalk W. (1984). The origin of the potato - an open problem. Nucleus 27, 37–44.

[B44] Hardigan M. A. Laimbeer F. P. E. Newton L. Crisovan E. Hamilton J. P. Vaillancourt B.. (2017). Genome diversity of tuber-bearing *Solanum* uncovers complex evolutionary history and targets of domestication in the cultivated potato. Proc. Natl. Acad. Sci. U.S.A. 114, E9999–E10008. doi: 10.1073/pnas.1714380114 29087343 PMC5699086

[B45] Hawkes J. G. (1956). Taxonomic studies on the tuber-bearing solanums. 1: *Solanum tuberosum* and the tetraploid species complex. Proc. Linn. Soc Lond. 166, 97–144. doi: 10.1111/j.1095-8312.1956.tb00754.x

[B46] Hayes M. M. MacIntyre A. M. Allen C. (2017). Complete genome sequences of the plant pathogens *Ralstonia solanacearum* type strain K60 and *R. solanacearum* race 3 biovar 2 strain UW551. Genome Announc. 5, e01088–e01117. doi: 10.1128/genomeA.01088-17 28983002 PMC5629059

[B47] Hayward A. C. (1964). Characteristics of *Pseudomonas solanacearum* . J. Appl. Bacteriol. 27, 265–277. doi: 10.1111/j.1365-2672.1964.tb04912.x

[B48] Hayward A. C. (1991). Biology and epidemiology of bacterial wilt caused by *Pseudomonas solanacearum* . Annu. Rev. Phytopathol. 29, 65–87. doi: 10.1146/annurev.py.29.090191.000433 18479193

[B49] Hayward A. C. (1994). “The hosts of pseudomonas solanacearum,” in Bacterial wilt: The disease and its causative agent, pseudomonas solanacearum. Eds. Hayward A. C. Hartman G. L. (Wallingford: CAB International), 9–24.

[B50] Hernández-Blanco C. Feng D. X. Hu J. Sánchez-Vallet A. Deslandes L. Llorente F.. (2007). Impairment of cellulose synthases required for *Arabidopsis* secondary cell wall formation enhances disease resistance. Plant Cell 19, 890–903. doi: 10.1105/tpc.106.048058 17351116 PMC1867366

[B51] Hirsch J. Deslandes L. Feng D. X. Balagué C. Marco Y. (2002). Delayed symptom development in *ein2-1*, an *Arabidopsis* ethylene-insensitive mutant, in response to bacterial wilt caused by *Ralstonia solanacearum* . Phytopathology 92, 1142–1148. doi: 10.1094/PHYTO.2002.92.10.1142 18944225

[B52] Höch K. Koopmann B. von Tiedemann A. (2021). Lignin composition and timing of cell wall lignification are involved in *Brassica napus* resistance to stem rot caused by *Sclerotinia sclerotiorum* . Phytopathology 111, 1438–1448. doi: 10.1094/PHYTO-09-20-0425-R 33386067

[B53] Hsu F. C. Chou M.-Y. Chou S.-J. Li Y.-R. Peng H.-P. Shih M.-C. (2013). Submergence confers immunity mediated by the WRKY22 transcription factor in *Arabidopsis* . Plant Cell 25, 2699–2713. doi: 10.1105/tpc.113.114447 23897923 PMC3753392

[B54] Huet G. (2014). Breeding for resistances to *Ralstonia solanacearum* . Front. Plant Sci. 5. doi: 10.3389/fpls.2014.00715 PMC4264415 25566289

[B55] Hussain A. Li X. Weng Y. Liu Z. Ashraf M. F. Noman A.. (2018). CaWRKY22 acts as a positive regulator in pepper response to *Ralstonia solanacearum* by constituting networks with CaWRKY6, CaWRKY27, CaWRKY40, and CaWRKY58. Int. J. Mol. Sci. 19, 1426. doi: 10.3390/ijms19051426 29747470 PMC5983767

[B56] Ishihara T. Mitsuhara I. Takahashi H. Nakaho K. (2012). Transcriptome analysis of quantitative resistance-specific response upon *Ralstonia solanacearum* infection in tomato. PloS One 7, e46763. doi: 10.1371/journal.pone.0046763 23071630 PMC3465262

[B57] Jackson M. T. (2012) Cruza 148. the serendipity of disease resistance. Available at: https://mikejackson1948.blog/2012/02/04/cruza-148-the-serendipity-of-disease-resistance/.

[B58] Jackson M. T. González L. C. Aguilar J. A. (1979). Avances en el combate de la marchitez bacteriana de papa en Costa Rica. Fitopatología 14, 46–53.

[B59] Jackson M. T. Rowe P. R. Hawkes J. G. (1978). Crossability relationships of Andean potato varieties of three ploidy levels. Euphytica 27, 541–551. doi: 10.1007/BF00043180

[B60] Janse J. D. Arulappan F. A. X. Schans J. Wenneker M. Westerhuis W. (1998). “Experiences with bacterial brown rot ralstonia solanacearum biovar 2, race 3 in the Netherlands,” in Bacterial wilt disease - molecular and ecological aspects. Eds. Prior P. Allen C. Elphinstone J. (Berlin-Heidelberg: Springer), 146–152.

[B61] Janse J. D. van den Beld H. E. Elphinstone J. Simpkins S. Tjou-Tam-Sin N. N. A. van Vaerenbergh J. (2004). Introduction to Europe of *Ralstonia solanacearum* biovar 2, race 3 in *Pelargonium zonale* cuttings. J. Plant Pathol. 86, 147–155.

[B62] Jaworski C. A. Webb R. E. Goth R. W. Phatak S. C. (1980). Relative resistance of potato cultivars to bacterial wilt. Am. Potato J. 57, 159–165. doi: 10.1007/BF02853867

[B63] Joly N. Souidi K. Depraetere D. Wils D. Martin P. (2021). Potato by-products as a source of natural chlorogenic acids and phenolic compounds: extraction, characterization, and antioxidant capacity. Molecules 26, 177. doi: 10.3390/molecules26010177 PMC7796066 33396560

[B64] Journot-Catalino N. Somssich I. E. Roby D. Kroj T. (2006). The transcription factors WRKY11 and WRKY17 act as negative regulators of basal resistance in *Arabidopsis thaliana* . Plant Cell 18, 3289–3302. doi: 10.1105/tpc.106.044149 17114354 PMC1693958

[B65] Kabir F. Katayama S. Tanji N. Nakamura S. (2014). Antimicrobial effects of chlorogenic acid and related compounds. J. Korean Soc Appl. Biol. 57, 359–365. doi: 10.1007/s13765-014-4056-6

[B66] Kanehisa M. Goto S. (2000). KEGG: Kyoto encyclopedia of genes and genomes. Nucl. Acids Res. 28, 27–30. doi: 10.1093/nar/28.1.27 10592173 PMC102409

[B67] Karim Z. Hossain M. S. Begum M. M. (2018). *Ralstonia solanacearum*: A threat to potato production in Bangladesh. Fund. Appl. Agric. 3, 407–421. doi: 10.5455/faa.280361

[B68] Kawamura Y. Hase S. Takenaka S. Kanayama Y. Yoshioka H. Kamoun S.. (2009). INF1 elicitin activates jasmonic acid- and ethylene-mediated signalling pathways and induces resistance to bacterial wilt disease in tomato. J. Phytopathol. 157, 287–297. doi: 10.1111/j.1439-0434.2008.01489.x

[B69] Kim K.-C. Fan B. Chen Z. (2006). Pathogen-induced *Arabidopsis WRKY7* is a transcriptional repressor and enhances plant susceptibility to *Pseudomonas syringae* . Plant Physiol. 142, 1180–1192. doi: 10.1104/pp.106.082487 16963526 PMC1630724

[B70] Kim D. Langmead B. Salzberg S. L. (2015). HISAT: a fast spliced aligner with low memory requirements. Nat. Methods 12, 357–360. doi: 10.1038/nmeth.3317 25751142 PMC4655817

[B71] Kim-Lee H. Moon J. S. Hong Y. J. Kim M. S. Cho H. M. (2005). Bacterial wilt resistance in the progenies of the fusion hybrids between haploid of potato and *Solanum commersonii* . Am. J. Potato Res. 82, 129–137. doi: 10.1007/BF02853650

[B72] Kinyua Z. M. Miller S. A. Chin A. Subedi N. (2014) Bacterial wilt disease ralstonia solanacearum: Standard operating procedure for use in diagnostic laboratories. Available at: https://ipmil.cired.vt.edu/wp-content/uploads/2014/06/SOP-Ralstonia-solanacerum-EastAfricaFinal-Apr2014-2.pdf.

[B73] Kong H. G. Bae J. Y. Lee H. J. Joo H. J. Jung E.J. Chung E.. (2014). Induction of the viable but nonculturable state of *Ralstonia solanacearum* by low temperature in the soil microcosm and its resuscitation by catalase. PloS One 9, e109792. doi: 10.1371/journal.pone.0109792 25296177 PMC4190316

[B74] Kunze G. Zipfel C. Robatsek S. Niehaus K. Boller T. Felix G. (2004). The n terminus of bacterial elongation factor tu elicits innate immunity in arabidopsis plants. Plant Cell 16, 3496–3507. doi: 10.1105/tpc.104.026765 15548740 PMC535888

[B75] Kurabachew H. Ayana G. (2016). Bacterial wilt caused by *Ralstonia solanacearum* in Ethiopia: Status and management approaches. Int. J. Phytopathol. 5, 107–119. doi: 10.33687/phytopath.005.03.1829

[B76] Lacombe S. Rougon-Cardoso A. Sherwood E. Peeters N. Dahlbeck D. van Esse H. P.. (2010). Interfamily transfer of a plant pattern-recognition receptor confers broad-spectrum bacterial resistance. Nat. Biotechnol. 28, 365–369. doi: 10.1038/nbt.1613 20231819

[B77] Laferriere L. T. Helgeson J. P. Allen C. (1999). Fertile *Solanum tuberosum* + *S. commersonii* somatic hybrids as sources of resistance to bacterial wilt caused by *Ralstonia solanacearum* . Theor. Appl. Genet. 98, 1272–1278. doi: 10.1007/s001220051193

[B78] Lang J. Colcombet J. (2020). Sustained incompatibility between MAPK signaling and pathogen effectors. Int. J. Mol. Sci. 21, 7954. doi: 10.3390/ijms21217954 33114762 PMC7672596

[B79] Liao Y. Smyth G. K. Shi W. (2014). featureCounts: an efficient general purpose program for assigning sequence reads to genomic features. Bioinformatics 30, 923–930. doi: 10.1093/bioinformatics/btt656 24227677

[B80] Liu Q. Luo L. Zheng L. (2018). Lignins: Biosynthesis and biological functions in plants. Int. J. Mol. Sci. 19, 335. doi: 10.3390/ijms19020335 29364145 PMC5855557

[B81] Liu T. Yu Y. Cai X. Tu W. Xie C. Liu J. (2016). Introgression of bacterial wilt resistance from *Solanum melongena* to *S. tuberosum* through asymmetric protoplast fusion. Plant Cell. Tissue Organ Cult. 125, 433–443. doi: 10.1007/s11240-016-0958-9

[B82] Livak K. J. Schmittgen T. D. (2001). Analysis of relative gene expression data using real-time quantitative PCR and the ^2–ΔΔ^CT method. Methods 25, 402–408. doi: 10.1006/meth.2001.1262 11846609

[B83] Li Y. Y. Wang L. Sun G. W. Li X. H. Chen Z. G. Feng J.. (2021). Digital gene expression analysis of the response to *Ralstonia solanacearum* between resistant and susceptible tobacco varieties. Sci. Rep. 11, 3887. doi: 10.1038/s41598-021-82576-8 33594109 PMC7886896

[B84] Long Q. Du M. Long J. Yu X. Zhang J. Xu L.. (2021). Transcription factor WRKY22 regulates canker susceptibility in sweet orange (*Citrus sinensis* osbeck) by enhancing cell enlargement and *CsLOB1* expression. Horticult. Res. 8, 50. doi: 10.1038/s41438-021-00486-2 PMC7917094 33642585

[B85] Love M. I. Huber W. Anders S. (2014). Moderated estimation of fold change and dispersion for RNA-seq data with DESeq2. Genome Biol. 15, 550. doi: 10.1016/j.tim.2018.06.002 25516281 PMC4302049

[B86] Lowe-Power T.M. Khokhani D. Allen C. (2018). How Ralstonia solanacearum exploits and thrives in the flowing plant xylem environment. Trends Microbiol. 26, 929–942. doi: 10.1016/j.tim.2018.06.002 29941188

[B87] Mansfield J. Genin S. Magori S. Citovsky V. Sriariyanum M. Ronald P.. (2012). Top 10 plant pathogenic bacteria in molecular plant pathology. Mol. Plant Pathol. 13, 614–629. doi: 10.1111/j.1364-3703.2012.00804.x 22672649 PMC6638704

[B88] Marin J. Battistuzzi F. U. Brown A. C. Hedges S. B. (2017). The timetree of prokaryotes: new insights into their evolution and speciation. Mol. Biol. Evol. 34, 437–446. doi: 10.1093/molbev/msw245 27965376

[B89] Martin C. (1979). “Sources of resistance to pseudomonas solanacearum,” in Developments in control of potato bacterial diseases (Lima: International Potato Center), 49–54.

[B90] Martin C. French E. R. (1985). “Pseudomonas solanacearum. technical information bulletin no. 13. sect. 2,” in Bacterial wilt of potato, vol. 8. (Lima: International Potato Center).

[B91] McGarvey J. A. Denny T. P. Schell M. A. (1999). Spatial-temporal and quantitative analysis of growth and EPS I production by *Ralstonia solanacearum* in resistant and susceptible tomato cultivars. Phytopathology 89, 1233–1239. doi: 10.1094/PHYTO.1999.89.12.1233 18944650

[B92] Meline V. Hendrich C. G. Truchon A. N. Caldwell D. Hiles R. Leuschen-Kohl R.. (2022). Tomato deploys defence and growth simultaneously to resist bacterial wilt disease. Plant Cell Environ. doi: 10.1111/pce.14456 36213953

[B93] Menna A. Dora S. Sancho-Andrés G. Kashyap A. Meena M. K. Sklodowski K.. (2021). A primary cell wall cellulose-dependent defense mechanism against vascular pathogens revealed by time-resolved dual transcriptomics. BMC Biol. 19, 161. doi: 10.1186/s12915-021-01100-6 34404410 PMC8371875

[B94] Miedes E. Vanholme R. Boerjan W. Molina A. (2014). The role of the secondary cell wall in plant resistance to pathogens. Front. Plant Sci. 5. doi: 10.3389/fpls.2014.00358 PMC4122179 25161657

[B95] Montanelli C. Stefanini F. M. Chiari A. Chiari T. Nascari G. (1995). Variability in the response to *Pseudomonas solanacearum* of transgenic lines of potato carrying a cecropin gene analogue. Potato Res. 38, 371–378. doi: 10.1007/BF02357742

[B96] Murashige T. Skoog F. (1962). A revised medium for rapid growth and bioassays with tobacco tissue cultures. Physiol. Plant. 15, 473–497. doi: 10.1111/j.1399-3054.1962.tb08052.x

[B97] Muthoni J. Shimelis H. Melis R. (2020). Conventional breeding of potatoes for resistance to bacterial wilt (Ralstonia solanacearum): Any light in the horizon? Austr. J. Crop Sci. 14, 485–494. doi: 10.21475/ajcs.20.14.03.p2144

[B98] Narancio R. Zorrilla P. Robello C. Gonzalez M. Vilaró F. Pritsch C.. (2013). Insights on gene expression response of a characterized resistant genotype of *Solanum commersonii* dun. against ralstonia solanacearum. Eur. J. Plant Pathol. 136, 823–835. doi: 10.1007/s10658-013-0210-y

[B99] Nicot N. Hausman J. F. Hoffmann L. Evers D. (2005). Housekeeping gene selection for real-time RT-PCR normalization in potato during biotic and abiotic stress. J. Exp. Bot. 56, 2907–2914. doi: 10.1093/jxb/eri285 16188960

[B100] Nielsen L. W. Haynes F. L. (1960). Resistance in *Solanum tuberosum* to *Pseudomonas solanacearum* . Am. Potato J. 37, 260–267. doi: 10.1007/BF02855800

[B101] Pál M. Ivanovska B. Oláh T. Tajti J. Hamow K.Á. Szalai G.. (2019). Role of polyamines in plant growth regulation of *Rht* wheat mutants. Plant Physiol. Biochem. 137, 189–202. doi: 10.1016/j.plaphy.2019.02.013 30798173

[B102] Pan X. Chen J. Yang A. Zhao W. Xu T. Chen B.. (2021). Comparative transcriptome profiling reveals defense-related genes against *Ralstonia solanacearum* infection in tobacco. Front. Plant Sci. 12. doi: 10.3389/fpls.2021.767882 PMC8712766 34970284

[B103] Park S. Gupta R. Krishna R. Kim S. T. Lee D. Y. Hwang D.. (2016). Proteome analysis of disease resistance against *Ralstonia solanacearum* in potato cultivar CT206-10. Plant Pathol. J. 32, 25–32. doi: 10.5423/PPJ.OA.05.2015.0076 26889112 PMC4755672

[B104] Patil V. U. Gopal J. Singh B. P. (2012). Improvement for bacterial wilt resistance in potato by conventional and biotechnological approaches. Agr. Res. 1, 299–316. doi: 10.1007/s40003-012-0034-6

[B105] Peeters N. Carrére S. Anisimova M. Plener L. Cazalé A.-C. Genin S. (2013). Repertoire, unified nomenclature and evolution of the type III effector gene set in the *Ralstonia solanacearum* species complex. BMC Genomics 14, 859. doi: 10.1186/1471-2164-14-859 24314259 PMC3878972

[B106] Peng J. Wang P. Fang H. Zheng J. Zhong C. Yang Y.. (2021). Weighted gene co-expression analysis network-based analysis on the candidate pathways and hub genes in eggplant bacterial wilt-resistance: a plant research study. Int. J. Mol. Sci. 22, 13279. doi: 10.3390/ijms222413279 34948076 PMC8706084

[B107] Pertea M. Pertea G. M. Antonescu C. M. Chang T. C. Mendell J. T. Salzberg S. L. (2015). StringTie enables improved reconstruction of a transcriptome from RNA-seq reads. Nat. Biotechnol. 3, 290–295. doi: 10.1038/nbt.3122 PMC4643835 25690850

[B108] Pfaffl M. W. (2004). “Quantification strategies in real-time PCR,” in A–z of quantitative PCR. Ed. Bustin S. A. (La Jolla: International University Line), 87–112.

[B109] Pham G. M. Hamilton J. P. Wood J. C. Burke J. T. Zhao H. Vaillancourt B.. (2020). Construction of a chromosome-scale long-read reference genome assembly for potato. GigaScience 9, giaa100. doi: 10.1093/gigascience/giaa100 32964225 PMC7509475

[B110] Planas-Marquès M. Kressin J. P. Kashyap A. Panthee D. R. Louws F. J. Coll N. S.. (2020). Four bottlenecks restrict colonization and invasion by the pathogen *Ralstonia solanacearum* in resistant tomato. J. Exp. Bot. 71, 2157–2171. doi: 10.1093/jxb/erz562 32211785 PMC7242079

[B111] Priou S. Salas C. De Mendiburu F. Aley P. Gutarra L. (2001). Assessment of latent infection frequency in progeny tubers of advanced potato clones resistant to bacterial wilt: A new selection criterion. Potato Res. 44, 359–373. doi: 10.1007/BF02358596

[B112] Ramos R. N. Martin G. B. Pombo M. A. Rosli H. G. (2021). WRKY22 and WRKY25 transcription factors are positive regulators of defense responses in *Nicotiana benthamiana* . Plant Mol. Biol. 105, 65–82. doi: 10.1007/s11103-020-01069-w 32909182

[B113] Särkinen T. Bohs L. Olmstead R. G. Knapp S. (2013). A phylogenetic framework for evolutionary study of the nightshades (Solanaceae): a dated 1000-tip tree. BMC Evol. Biol. 13, 214. doi: 10.1186/1471-2148-13-214 24283922 PMC3850475

[B114] Savary S. Willocquet L. Pethybridge S. J. Esker P. McRoberts N. Nelson A. (2019). The global burden of pathogens and pests on major food crops. Nat. Ecol. Evol. 3, 430–443. doi: 10.1038/s41559-018-0793-y 30718852

[B115] Schmiediche P. (1983). “Breeding bacterial wilt (Pseudomonas solanacearum) resistant germplasm,” in Present and future strategies for potato breeding and improvement (Lima: International Potato Center), 45–55.

[B116] Sequeira L. Rowe P. R. (1969). Selection and utilization of *Solanum phureja* clones with high resistance to different strains of *Pseudomonas solanacearum* . Am. Potato J. 46, 451–462. doi: 10.1007/BF02862028

[B117] Sheikh A. H. Hussain R. M. F. Tabassum N. Badmi R. Marillonnet S. Scheel D.. (2021). Possible role of WRKY transcription factors in regulating immunity in oryza sativa ssp. *indica* . Physiol. Mol. Plant Pathol. 114, 101623. doi: 10.1016/j.pmpp.2021.101623

[B118] Silva N. Mazzafera P. Cesarino I. (2019). Should I stay or should I go: are chlorogenic acids mobilized towards lignin biosynthesis? Phytochemistry 166, 112063. doi: 10.1016/j.phytochem.2019.112063 31280091

[B119] Siri M. I. Galván G. Quirici L. Silvera E. Villanueva P. Ferreira F. (2009). Molecular marker diversity and bacterial wilt resistance in wild Solanum commersonii accessions from Uruguay. Euphytica 165, 371–382. doi: 10.1007/s10681-008-9800-8

[B120] Spooner D. M. McLean K. Ramsay G. Waugh R. Bryan G. J. (2005). A single domestication for potato based on multilocus amplified fragment length polymorphism genotyping. Proc. Natl. Acad. Sci. U.S.A. 102, 14694–14699. doi: 10.1073/pnas.0507400102 16203994 PMC1253605

[B121] Stevenson F. J. Akeley R. V. Newton G. Isleib D. (1965). Monona: A new variety of potato, distinctive for excellent chip color after numerous storage treatments. Am. Potato J. 42, 253–255. doi: 10.1007/BF02861153

[B122] Stiekema W. J. Heidekamp F. Dirkse W. G. van Beckum J. de Haan P. Bosch C. T.. (1988). Molecular cloning and analysis of four potato tuber mRNAs. Plant Mol. Biol. 11, 255–269. doi: 10.1007/BF00027383 24272339

[B123] Swanson J. K. Yao J. Tans-Kersten J. Allen C. (2005). Behavior of *Ralstonia solanacearum* race 3 biovar 2 during latent and active infection of geranium. Phytopathology 95, 136–143. doi: 10.1094/PHYTO-95-0136 18943982

[B124] Thurston H. D. Lozano J. C. (1968). Resistance to bacterial wilt of potatoes in Colombian clones of *Solanum phureja* . Am. Potato J. 45, 51–55. doi: 10.1007/BF02862862

[B125] Tung P. X. Rasco E. T. Vander Zaag P. Schmiediche P. (1990). Resistance to *Pseudomonas solanacearum* in the potato: I. effects of sources of resistance and adaptation. Euphytica 45, 203–210. doi: 10.1007/BF00032987

[B126] Tyagi B. R. Mishra P. C. Shekhawat G. S. Singh R. (1980). Transfer of brown rot resistance from *Solanum microdontum* to *S. tuberosum* . J. Indian Potato Assoc. 7, 192–195.

[B127] Valiñas M. A. Lanteri M. L. ten Have A. Andreu A. B. (2015). Chlorogenic acid biosynthesis appears linked with suberin production in potato tuber (*Solanum tuberosum*). J. Agric. Food Chem. 63, 4902–4913. doi: 10.1021/jf505777p 25921651

[B128] van Elsas J. D. Kastelein P. van Bekkum P. van der Wolf J. M. de Vries P. M. van Overbeek L. S. (2000). Survival of *Ralstonia solanacearum* biovar 2, the causative agent of potato brown rot, in field and microcosm soils in temperate climates. Phytopathology 90, 1358–1366. doi: 10.1094/PHYTO.2000.90.12.1358 18943377

[B129] Vasse J. Frey P. Trigalet A. (1995). Microscopic studies of intercellular infection and protoxylem invasion of tomato roots by *Pseudomonas solanacearum* . Mol. Plant-Microbe Int. 8, 241–251. doi: 10.1094/MPMI-8-0241

[B130] Verma V. Ravindran P. Kumar P. P. (2016). Plant hormone-mediated regulation of stress responses. BMC Plant Biol. 16, 86. doi: 10.1186/s12870-016-0771-y 27079791 PMC4831116

[B131] Vrhovsek U. Masuero D. Gasperotti M. Franceschi P. Caputi L. Viola R.. (2012). A versatile targeted metabolomics method for the rapid quantification of multiple classes of phenolics in fruits and beverages. J. Agric. Food Chem. 60, 8831–8840. doi: 10.1021/jf2051569 22468648

[B132] Wang D. Amornsiripanitch N. Dong X. (2006). A genomic approach to identify regulatory nodes in the transcriptional network of systemic acquired resistance in plants. PloS Pathog. 2, e123. doi: 10.1371/journal.ppat.0020123 17096590 PMC1635530

[B133] Wang H. Cheng Z. Wang B. Dong J. Ye W. Yu Y.. (2020). Combining genome composition and differential gene expression analyses reveals that *SmPGH1* contributes to bacterial wilt resistance in somatic hybrids. Plant Cell Rep. 39, 1235–1248. doi: 10.1007/s00299-020-02563-7 32666195

[B134] Wang K. Kang L. Anand A. Lazarovits G. Mysore K. S. (2007). Monitoring in planta bacterial infection at both cellular and whole-plant levels using the green fluorescent protein variant GFPuv. New Phytol. 174, 212–223. doi: 10.1111/j.1469-8137.2007.01999.x 17335510

[B135] Wan J. He M. Hou Q. Zou L. Yang Y. Wei Y.. (2021). Cell wall associated immunity in plants. Stress Biol. 1, 3. doi: 10.1007/s44154-021-00003-4 PMC10429498 37676546

[B136] Wei Y. Zhang Y. Meng J. Wang Y. Zhong C. Ma H. (2021). Transcriptome and metabolome profiling in naturally infested *Casuarina equisetifolia* clones by *Ralstonia solanacearum* . Genomics 113, 1906–1918. doi: 10.1016/j.ygeno.2021.03.022 33771635

[B137] Wu T. Hu E. Xu S. Chen M. Guo P. Dai Z.. (2021). ClusterProfiler 4.0: A universal enrichment tool for interpreting omics data. Innovation (Camb.) 2, 100141. doi: 10.1016/j.xinn.2021.100141 34557778 PMC8454663

[B138] Xiao X. Cao B. Li G. Lei J. Chen Q. Jiang J.. (2015). Functional characterization of a putative bacterial wilt resistance gene (*RE-bw*) in eggplant. Plant Mol. Biol. Rep. 33, 1058–1073. doi: 10.1007/s11105-014-0814-1

[B139] Xue H. Lozano-Durán R. Macho A. P. (2020). Insights into the root invasion by the plant pathogenic bacterium *Ralstonia solanacearum* . Plants 9, 516. doi: 10.3390/plants9040516 32316375 PMC7238422

[B140] Yadav V. Wang Z. Wei C. Amo A. Ahmed B. Yang X.. (2020). Phenylpropanoid pathway engineering: an emerging approach towards plant defense. Pathogens 9, 312. doi: 10.3390/pathogens9040312 32340374 PMC7238016

[B141] Yin Z. Xie F. Michalak K. Pawełkowicz M. Zhang B. Murawska Z.. (2016). Potato cultivar etola exhibits hypersensitive resistance to PVY^NTN^ and partial resistance to PVY^Z-NTN^ and PVY^N-wi^ strains and strain-specific alterations of certain host miRNAs might correlate with symptom severity. Plant Pathol. 6, 539–550. doi: 10.1111/ppa.12599

[B142] Yu Y. Ye W. He L. Cai X. Liu T. Liu J. (2013). Introgression of bacterial wilt resistance from eggplant to potato *via* protoplast fusion and genome components of the hybrids. Plant Cell Rep. 32, 1687–1701. doi: 10.1007/s00299-013-1480-8 23912850

[B143] Zaynab M. Fatima M. Abbas S. Sharif Y. Umair M. Zafar M. H.. (2018). Role of secondary metabolites in plant defense against pathogens. Microb. Pathog. 124, 198–202. doi: 10.1016/j.micpath.2018.08.034 30145251

[B144] Zhang C. Chen H. Cai T. Deng Y. Zhuang R. Zhang N.. (2017). Overexpression of a novel peanut *NBS-LRR* gene *AhRRS5* enhances disease resistance to *Ralstonia solanacearum* in tobacco. Plant Biotechnol. J. 15, 39–55. doi: 10.1111/pbi.12589 27311738 PMC5253469

[B145] Zhao C. Wang H. Lu Y. Hu J. Qu L. Li Z.. (2019). Deep sequencing reveals early reprogramming of *Arabidopsis* root transcriptomes upon *Ralstonia solanacearum* infection. Mol. Plant-Microbe Int. 31, 813–827. doi: 10.1094/MPMI-10-18-0268-R 31140930

[B146] Zipfel C. Kunze G. Chinchilla D. Caniard A. Jones J. D. G. Boller T.. (2006). Perception of the bacterial PAMP EF-tu by the receptor EFR restricts *Agrobacterium*-mediated transformation. Cell 125, 749–760. doi: 10.1016/j.cell.2006.03.037 16713565

[B147] Zuluaga A. P. Solé M. Lu H. Góngora-Castillo E. Vaillancourt B. Coll N.. (2015). Transcriptome responses to *Ralstonia solanacearum* infection in the roots of the wild potato *Solanum commersonii* . BMC Genomics 16, 246. doi: 10.1186/s12864-015-1460-1 25880642 PMC4391584

