# Supplementary material for: Global transcriptome and targeted metabolite analyses of roots reveal different defence mechanisms against Ralstonia solanacearum infection in two resistant potato cultivars
Source: Front Plant Sci. 2023 Jan 9;13:1065419. doi: 10.3389/fpls.2022.1065419 (PMC9889091; doi:10.3389/fpls.2022.1065419)
Supplement: Supplementary file 1 [file DataSheet_1.zip › FigS1.pdf]

Fig. S1

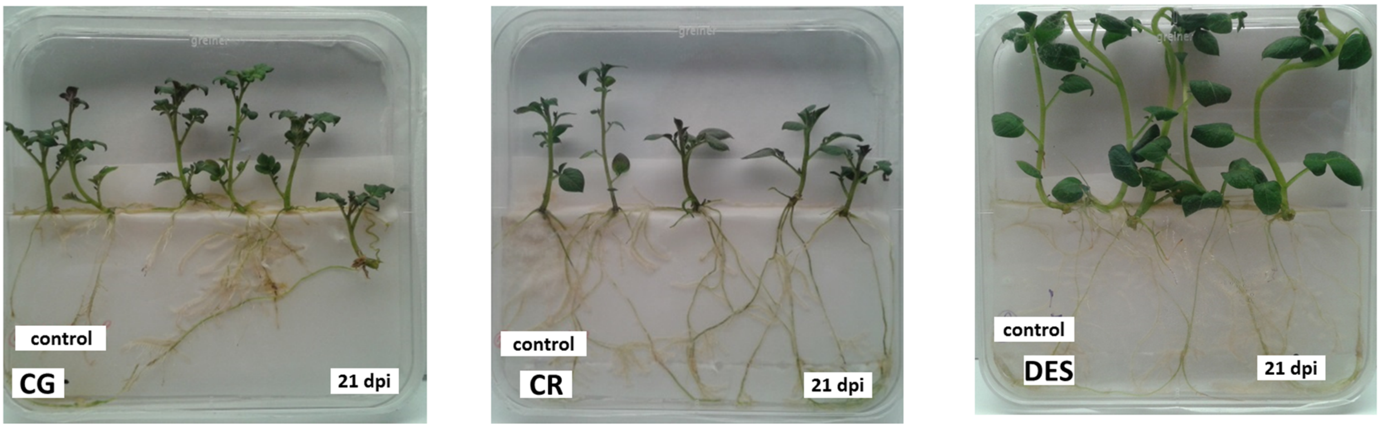

Fig. S1. Noninfected control plants grown *in vitro* in parallel with *Rs*-infected (21 dpi) plants (see Figure 1) of ‛Calalo Gaspar’ (CG), ‛Cruza 148’ (CR), and ‛Désirée’ (DES).
