# Supplementary material for: Global transcriptome and targeted metabolite analyses of roots reveal different defence mechanisms against Ralstonia solanacearum infection in two resistant potato cultivars
Source: Front Plant Sci. 2023 Jan 9;13:1065419. doi: 10.3389/fpls.2022.1065419 (PMC9889091; doi:10.3389/fpls.2022.1065419)
Supplement: Supplementary file 1 [file DataSheet_1.zip › FigS2.pdf]

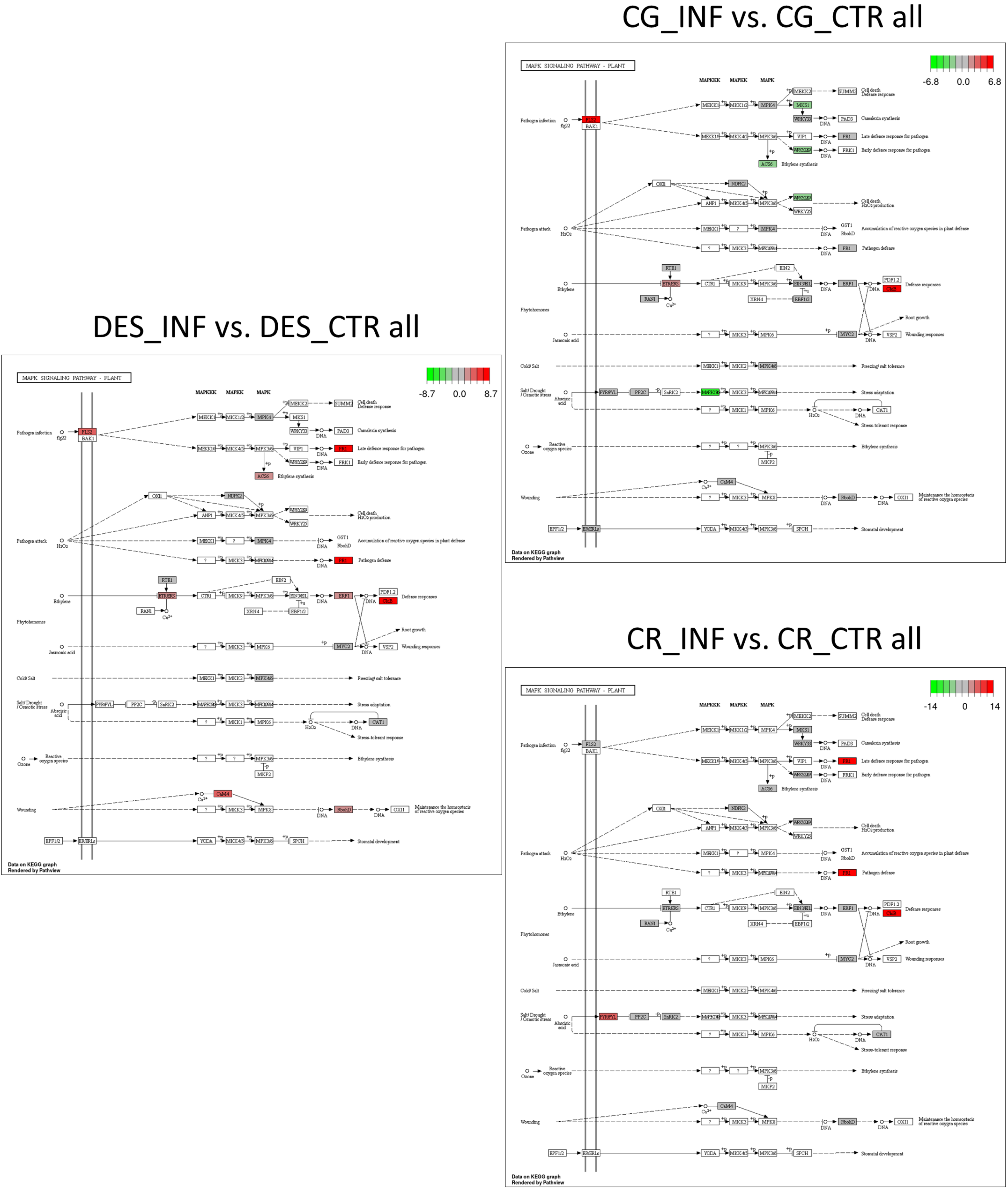

Fig. S2. MAPK signalling pathway sot04016. DEGs of the pathway in *Rs*-infected (2 dpi) roots 'Désirée' (DES), 'Calalo Gaspar' (CG), and 'Cruza 148' (CR) are coloured. INF, infected; CTR, noninfected control
