# Supplementary material for: Global transcriptome and targeted metabolite analyses of roots reveal different defence mechanisms against Ralstonia solanacearum infection in two resistant potato cultivars
Source: Front Plant Sci. 2023 Jan 9;13:1065419. doi: 10.3389/fpls.2022.1065419 (PMC9889091; doi:10.3389/fpls.2022.1065419)
Supplement: Supplementary file 1 [file DataSheet_1.zip › FigS5.pdf]

	'Calalo Gaspar' *Rs*-resistant	'Cruza 148' *Rs*-resistant	'Désirée' *Rs*-susceptible
**Gene ontology (GO) enrichment at 2 dpi in *Rs*-infected roots**			
photosystem-related genes	down	-	down
oxidative stress and stress response-related genes	down	-	-
cell wall-related genes	-	up	up
chitin interaction-related genes	-	up	up
carbohydrate metabolic process-related genes	-	down	-
**KEGG pathways at 2 dpi in *Rs*-infected roots**			
phenylpropanoid biosynthesis genes	down	up	-
plant-pathogen interaction-related genes	down	down	-
glutathione metabolism genes	-	up	-
MAPK signalling pathway genes	-	-	up
**Metabolite and plant hormone concentrations at 6 dpi in *Rs*-infected roots**			
significant differences in the concentration of phenolics and plant hormones between non-infected and *Rs*-infected roots	yes	no	yes
chlorogenic acid concentration in non-infected roots	high	medium	low
increased crypto-/neochlorogenic acid concentration in *Rs*-infected roots	yes	no	no
vanillin concentration in control and *Rs*-infected roots	high	low	low
syringaldehyde concentration in control and *Rs*-infected roots	high	low	low
quercetin derivatives concentration upon *Rs*-infection	high	low	low
jasmonic acid (JA) concentration in non-infected roots	-	-	detectable
increased JA concentration upon *Rs*-infection	-	-	yes
salicylic acid (SA) concentration in non-infected roots	medium	low	medium
increased SA concentration upon *Rs*-infection	yes	yes	yes (very high)
abscisic acid (ABA) concentration in non-infected roots	low	high	medium
increased ABA concentration upon *Rs*-infection	no	no	yes
indoleacetic acid (IAA) concentration in non-infected roots	low	high	low
increased IAA concentration upon *Rs*-infection	yes	no	yes
increased lignification upon *Rs*-infection based on confocal microscopy	no	yes (stele)	yes (xylem)

Fig. S5. Characteristic differences between the tested potato cultivars in their transcriptomic and metabolic reactions to *Rs* infection. (- indicates no effects detected)
