# Supplementary figures and images for: Global transcriptome and targeted metabolite analyses of roots reveal different defence mechanisms against Ralstonia solanacearum infection in two resistant potato cultivars
Source: Front Plant Sci. 2023 Jan 9;13:1065419. doi: 10.3389/fpls.2022.1065419 (PMC9889091; doi:10.3389/fpls.2022.1065419)

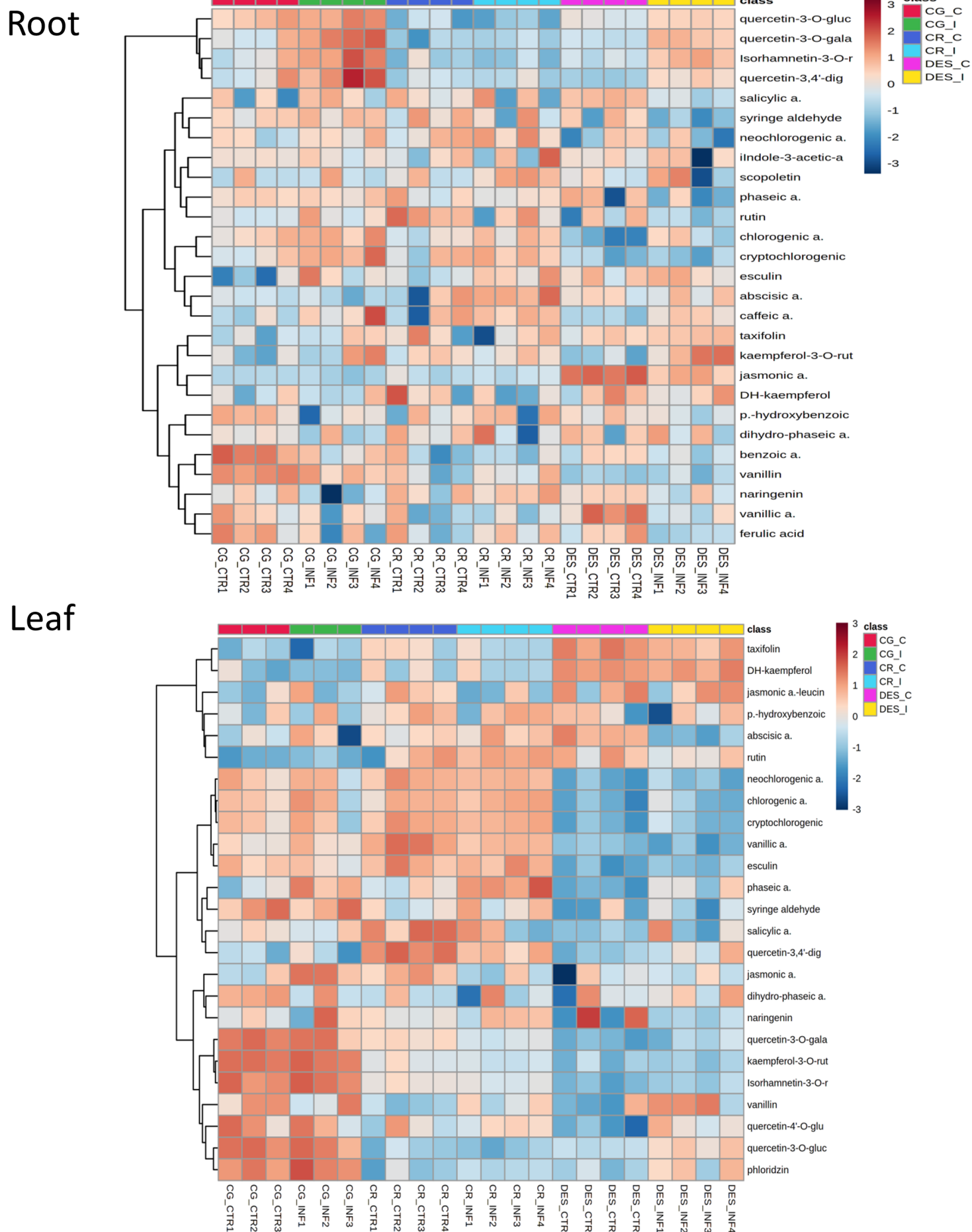

Supplement: Supplementary file 1 [file DataSheet_1.zip › FigS4.pdf]
